# Site Selective and Efficient Spin Labeling of Proteins with a Maleimide-Functionalized Trityl Radical for Pulsed Dipolar EPR Spectroscopy

**DOI:** 10.3390/molecules24152735

**Published:** 2019-07-27

**Authors:** J. Jacques Jassoy, Caspar A. Heubach, Tobias Hett, Frédéric Bernhard, Florian R. Haege, Gregor Hagelueken, Olav Schiemann

**Affiliations:** Institute of Physical and Theoretical Chemistry, Rheinische Friedrich-Wilhelms-University Bonn, Wegelerstr. 12, 53115 Bonn, Germany

**Keywords:** proteins, distance measurements, EPR, DQC, PELDOR, SIFTER

## Abstract

Pulsed dipolar electron paramagnetic resonance spectroscopy (PDS) in combination with site-directed spin labeling (SDSL) of proteins and oligonucleotides is a powerful tool in structural biology. Instead of using the commonly employed *gem*-dimethyl-nitroxide labels, triarylmethyl (trityl) spin labels enable such studies at room temperature, within the cells and with single-frequency electron paramagnetic resonance (EPR) experiments. However, it has been repeatedly reported that labeling of proteins with trityl radicals led to low labeling efficiencies, unspecific labeling and label aggregation. Therefore, this work introduces the synthesis and characterization of a maleimide-functionalized trityl spin label and its corresponding labeling protocol for cysteine residues in proteins. The label is highly cysteine-selective, provides high labeling efficiencies and outperforms the previously employed methanethiosulfonate-functionalized trityl label. Finally, the new label is successfully tested in PDS measurements on a set of doubly labeled *Yersinia* outer protein O (YopO) mutants.

## 1. Introduction

**Figure 1:**
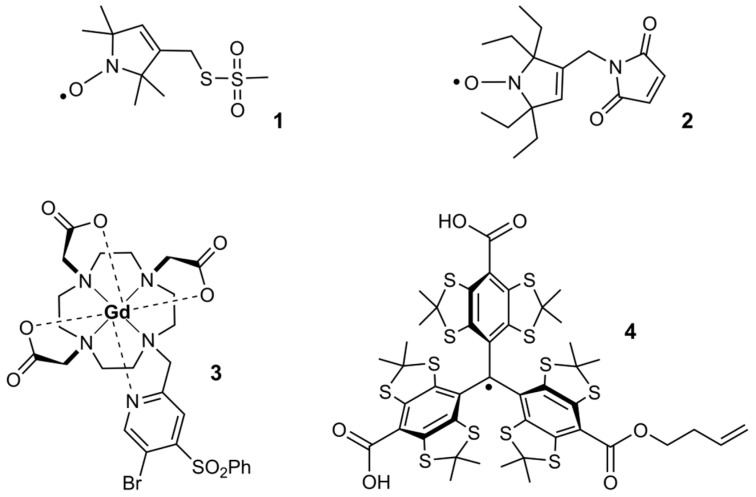
The molecular structures of selected spin labels: MTSSL **1**, M-TETPO **2**, BrPSPy-DO3A-Gd(III) **3**, TSL-BUTENE **4**.

The combination of site-directed spin labeling (SDSL) with electron paramagnetic resonance (EPR) spectroscopy has proven to be a valuable tool in structural biology [1,2,3]. In particular, the use of pulsed dipolar EPR spectroscopy (PDS) methods for measuring distances between spin centers in the range of 1.6–16 nm, like pulsed electron-electron double resonance (PELDOR or DEER) [4,5], the double quantum coherence experiment (DQC) [6,7,8], the single frequency technique for refocusing dipolar couplings (SIFTER) [9] or relaxation induced dipolar modulation enhancement (RIDME) [10,11] have been very successful in providing information on the structure, conformational changes and dynamics of proteins [12,13,14,15,16,17,18], oligonucleotides [19,20,21,22,23] and their complexes [24,25]. Most of these studies rely on spin labeling with nitroxides. For protein labeling, the most established spin label is the methanethiosulfonate-functionalized nitroxide MTSSL **1** (Figure 1), which reacts with cysteine residues to form the disulfide bonded side chain **R1** [26,27]. MTSSL provides high labeling yields and site selectivity through a combination with site-directed mutagenesis, which places the cysteines and thus the **R1** side chain at the desired positions in the protein.

In order to study biomolecules under physiological conditions, it would be highly desirable to perform such SDSL/PDS studies at room temperature in the liquid state and within the cells. However, such studies involving nitroxides as spin labels are usually limited to frozen buffer solutions due to the relaxation behavior of the nitroxides [27,28]. Furthermore, under *in cell* conditions, MTSSL, as well as all *gem*-dimethyl nitroxides, is quickly reduced to EPR-inactive hydroxylamine [29] and the bioconjugating MTSSL disulfide bond is reductively cleaved [30]. Thus, new cysteine targeting spin labels have been designed and tested *in cell* to address these issues: (a) Sterically shielded nitroxides such as **2** [31], (b) gadolinium(III)-based spin tags like **3** [32] and (c) triarylmethyl (trityl) radicals such as **4** (Figure 1) [33]. The compounds **2**–**4** are bioconjugated to cysteine residues via stable thioether bonds and show an increased in cell EPR signal persistency.

**Figure 2:**
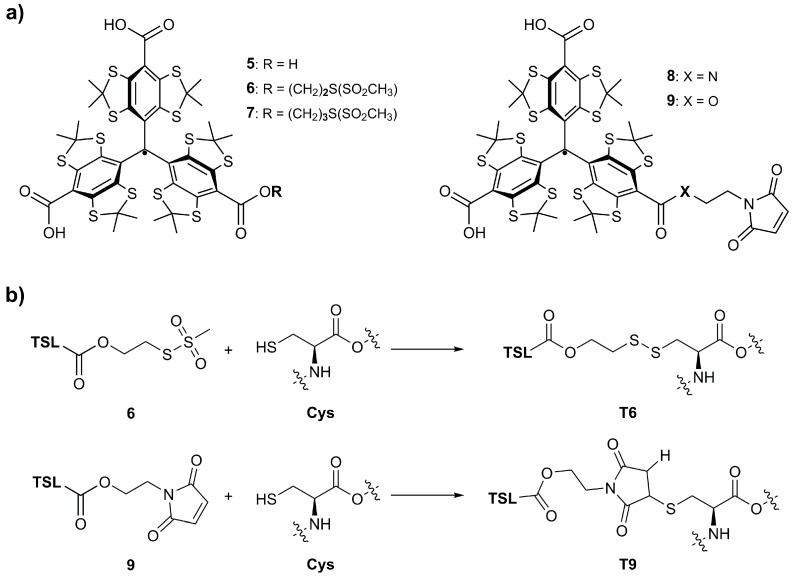
Lewis structures of Finland Trityl **5** and trityl spin labels **6**–**9** (**a**). Exemplary bioconjugation of trityl spin labels (TSL) **6** and **9** to cysteine residues resulting in the modified side chains **T6** and **T9** (**b**).

In particular, trityl spin labels hold great promise because they feature not only extended life times within the cells [34] but also several EPR spectroscopic distinctions from nitroxides and Gd-complexes, which can be advantageous in orthogonal spin labeling strategies [25,28,35,36,37]. Trityl spin labels based on the Finland Trityl **5** (Figure 2), display a single narrow line [38,39,40], which increases the EPR sensitivity and favors the use of single-frequency EPR experiments, such as SIFTER [9] or DQC [6,7,8]. Additionally, the carbon centered trityl spins show longer phase memory times *T_m_* at room temperature in the liquid state than paramagnetic metal or nitroxide spin centers [41,42], enabling pulsed EPR distance measurements at physiological temperatures [28,35,43,44,45].

Since the introduction of **5** [46] many synthesis improvements [33,47,48,49,50] and derivatization strategies [44,49,50,51,52,53,54,55,56,57,58,59] as well as applications of trityl compounds in medicinal probing [60,61], imaging [62,63], as magnetic materials [64], and as spin labels in structural biology [28,33,35,36,37,43,44,45,65] have been reported. Recent examples for the trityl labeling of cysteine residues in proteins used butene (**4**, Figure 1) [33] or methanethiosulfonate (**6** and **7**, Figure 2) [33,35,36] derivatives of **5** to establish the bioconjugation via thioether bonds for *in cell* studies or via disulfide bonds for in vitro studies, respectively. However, both approaches revealed complications, namely a low labeling efficiency of 36% in the case of the butene derivative **4** [33] and unspecific, non-covalent protein-trityl aggregation in the case of **5**, **7**, and **8** [36,65,66].

In order to further develop the scope of protein labeling with trityl radicals, this work presents the synthesis and characterization of the maleimide-derivatized trityl **9** (Figure 2) as well as a procedure for its selective bioconjugation to cysteine residues. Its labeling performance is meticulously assessed and compared to its predecessor **6** using *Yersinia* outer protein O mutants (YopO, ~72 kDa) [67,68] as a model system. The spin labels **8** and **9** differ with respect to the linker group, amide in the case of the former and ester for the latter. While the increased stability of amides against hydrolytic cleavage might be beneficial for *in cell* SDSL-EPR, the stronger electron withdrawing ester substituent in **9** was employed here to avert the reported EPR signal loss for **8**, due to oxidation of the trityl radical [49,65,69]. Finally, trityl label **9** is tested in trityl-trityl distance measurements by means of DQC, SIFTER and PELDOR experiments on doubly labeled YopO mutants and compared to the data obtained from the corresponding MTSSL-labeled protein.

## 2. Results and Discussion

### 2.1. Synthesis

The parent compound **5** was synthesized according to the literature [33,47,48,49] and esterified with the alcohol 2-hydroxyethyl maleimide using 2-chloro-1-methylpyridinium iodide (CMPI) as the activator [33,70]. After column chromatography, compound **9** was obtained as a brown solid in a yield of 21%. The identity of **9** was confirmed through ESI(+)-HRMS, UV/Vis, and *cw* EPR spectroscopy and its purity was assessed through MALDI-TOF mass spectrometry as well as medium pressure liquid chromatography (Figure S1–S7).

### 2.2. Redox-Stability of Trityls

**Figure 3:**
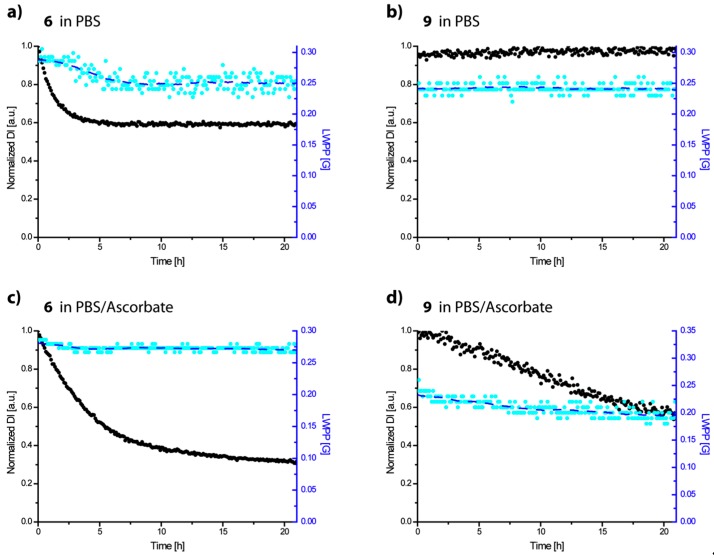
The stability of trityl labels **6** and **9**. The normalized double integral values (black), peak-to-peak line width values (cyan) of 200 µM gas tight incubations of **6** (**a**,**c**) and **9** (**b**,**d**) in PBS buffer and in PBS buffer with 5 mM ascorbate. EMXmicro acquisition parameters: modulation amplitude: 0.1 G, microwave power: 558 µW, time constant: 20.48 ms, sweep time: 69.02 s, resolution: 100 Pts/G for **6** and 154 Pts/G for **9**, sweep width: 20 G.

The chemical stability of the previously used methanethiosulfonate-trityl label **6** and the new maleimide-functionalized trityl label **9** was compared by monitoring their *cw* EPR spectra in gas-tightly sealed aqueous buffer solutions with and without ascorbate over a period of 21 h (Figure 3). In the absence of ascorbate, it was found that the double integral of **6** reduced to 60% of the initial amplitude after ~6 h before reaching a plateau level. During the same period, the line width reduced from 0.30 to 0.24 G. This finding points towards the oxygen consuming generation of diamagnetic trityl anions and the eventual stop of this reaction after all oxygen has been consumed (Figure 3a) [69]. In contrast, **9** shows stable double integral values and line widths under the same conditions (Figure 3b). In the presence of a 25-fold molar excess of ascorbate as a reducing agent, the double integral value of **6** is halved after 5 h (Figure 3c) whereas label **9** (Figure 3d) decayed only by 10% within the same time. Both set-ups demonstrate that trityl label **9** is considerably more redox-stable than **6**, which is beneficial for EPR experiments under the reducing conditions of *in cell* studies.

### 2.3. Labeling

In previous publications, it was found that trityl radicals derived from Finland Trityl **5** aggregate in aqueous solution above 60 µM [64] and that non-bioconjugated trityl remnants were often found next to trityl-labeled proteins even after separation attempts with size exclusion chromatography [36,65]. However, all reported trityl labeling procedures of the proteins used trityl concentrations in the range of 200–1500 µM [33,36,43,65], meaning that the formation of trityl aggregates was favored. Therefore, it was tested here, whether it is possible to suppress trityl aggregation and thus facilitate the separation of the excess label by working with free trityl label concentrations not far above the critical aggregation concentration during the bioconjugation and the subsequent purification steps.

**Figure 4:**
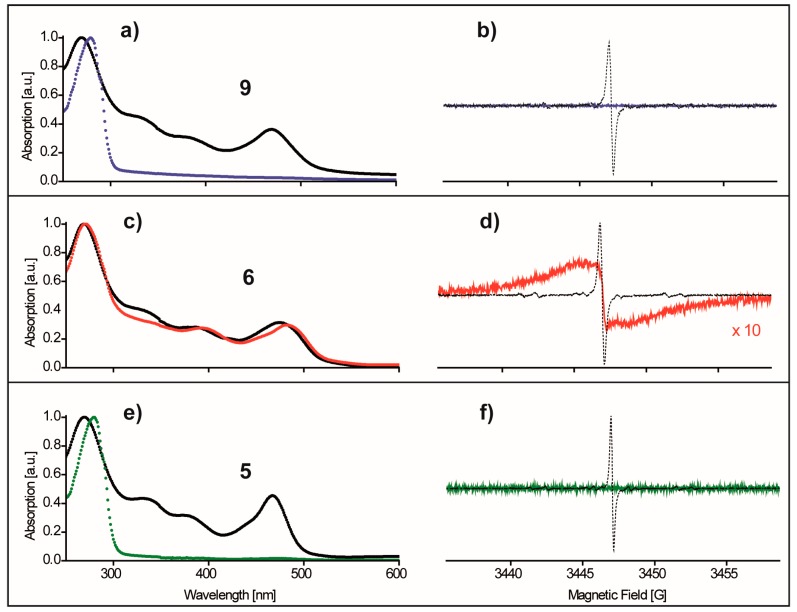
The incubations of **9** (blue), **6** (red) and **5** (green) with cysteine-free YopO-WT. The spectra shown have been recorded after size exclusion chromatography. Panels (**a**,**c**,**e**): normalized UV/Vis spectra of the protein solutions in juxtaposition with 20 µM buffer solutions of the pure trityl compounds **9**, **6**, and **5** (black). Panels (**b**,**d**,**f**): corresponding room temperature *cw* X-band EPR spectra overlaid with the spectrum of the free label (dashed black line) for the sake of comparison.

In a first test, the cysteine free protein construct YopO C219A (further on referred to as YopO-WT) was incubated with maleimide trityl **9**, methanethiosulfonate trityl **6** and the parent trityl **5** as a non-bioconjugatable reference benchmark. Since YopO-WT has no cysteines, none of the samples should show a trityl signal after incubation and the separation of the excess label via size exclusion chromatography. All incubations were performed in phosphate buffer solutions at pH = 6.8 in order to disfavor competing reactions of **9** with the 35 lysine residues [71] within YopO-WT and to avoid the deactivating hydrolysis of the bioconjugating maleimide moiety [72]. The trityl labels were prepared as 84 µM solutions in the buffer (2.50 mL) and added to the protein solutions (3.50 mL), resulting in final incubation concentrations of 35 µM and 3.5 µM for label and protein, respectively. After size exclusion chromatography (PD-10), the protein solutions were concentrated to approximately 5 µM and analyzed by UV/Vis spectroscopy and *cw* EPR (Figure 4).

According to the obtained UV/Vis and EPR spectra, excess label **9** (Figure 4a,b, blue trace) and **5** (Figure 4e,f, green trace) were successfully separated from the cysteine-free YopO-WT protein as seen by the absence of the characteristic trityl UV/Vis band at ~467 nm (black traces in Figure 4a,e), the absence of an EPR signal from these samples and mass spectrometry only detecting unlabeled protein masses for the incubation with **9** (Figure S14). In contrast, the incubation of YopO-WT with **6** lead even at this low trityl concentration and after column chromatography to a UV/Vis absorption band at ~467 nm and a significantly broadened trityl signal in the *cw* EPR spectrum (Figure 4c,d, red trace). This does clearly indicate that trityl remnants could not be separated from the protein in this case. Interestingly, such a broadened *cw* EPR spectrum was also obtained upon prolonged incubations (16 h, 4 °C) of **6** without the protein (Figure S16a). As no comparable line broadening was found for protein free incubations of **9** (Figure S16b) and **5**, the deviant properties of **6** must be related to its methanethiosulfonate moiety. As it is known that MTSSL **1** forms a disulfide-bridged bisnitroxide in solution over time [27], it can be reasonably assumed that also **6** forms such a disulfide-bridged bistrityl compound leading to the observed broadening of the EPR signal. This hypothesis was tested by irradiation of the sample from Figure S16a with UV light of λ = 254 nm in order to cleave the disulfide bridge [73], and to recover the corresponding narrow line EPR spectrum, which is exactly what was observed (Figure S17). At the same time, the spin count before and after UV light irradiation did not change, indicating that the line does not narrow due to a light-induced depletion of trityl centers. The attempts to disfavor the dimerization of **6** by varying the incubation time and temperatures did not help, nor was it possible to separate the seemingly aggregated dimers by using other chromatography methods (AEKTA, size exclusion and hydrophobic interaction materials) or dialysis procedures (20 h, ×5 million volume dilution, 5000 MWCO membrane). Remarkably, no evidence for free label remnants were reported in the labeling of the solid support fixated T4 lysozyme protein with **7** [35] and with another disulfide trityl derivative CT02-TP [43]. Apparently, the aggregated label as well as possible dimerization products could be removed from the protein sample by repeated washing of the protein-loaded beads with buffer solution. In the present work on YopO, similar attempts using ^6^His tag modified YopO mutants immobilized on nickel affinity beads did not lead to the separation of residual **6**.

Next, a general labeling procedure for the maleimide-derivatized label **9** was developed using YopO mutants S88C/L113C and YopO L113C/L353C with highly exposed cysteine residues under varied reaction conditions. In each case, the criterion in the evaluation of the labeling outcome was the resulting trityl/protein ratio after size exclusion chromatography as determined by UV/Vis spectroscopy (Supplementary Materials, Section 4.2). The following aspects turned out to be crucial for a successful labeling: (a) The trityl concentration in the labeling solution has to be kept below 35 µM until after the separation of the free label from the labeled protein. Otherwise, trityl aggregates were formed [64] that exceeded the molecular cut-off (MWCO = 5000) of the employed PD-10 size exclusion column and were eluted alongside the protein. (b) At the cost of prolonged reaction times (16 h), the labeling proceeded best in slightly acidic solutions (pH = 6.5–6.8) and at low temperatures (4 °C). In contrast, the attempt to increase the cysteine nucleophilicity through basic solution conditions (pH = 7.5–8.5) as usually done [65,74], resulted in excessive trityl/protein ratios that could indicate lysine labeling under alkaline conditions [71]. Increasing the temperature to room temperature led to lower labeling efficiencies, maybe due to the accelerated hydrolyzation of the maleimide group [72]. This competing process is conventionally countered by favoring the second order labeling reactions over the pseudo first order hydrolyzation reactions via high label concentrations, an option which is not possible in the present case. (c) The separation of the free label via PD-10 column worked best when the column was loaded with 2 mL of the incubation solution, i.e., 70 nmol of **9**, followed by 500 µL of buffer solution. The higher trityl amounts per loading apparently surpassed the column capacity and led to only partial removal of the free label.

**Figure 5:**
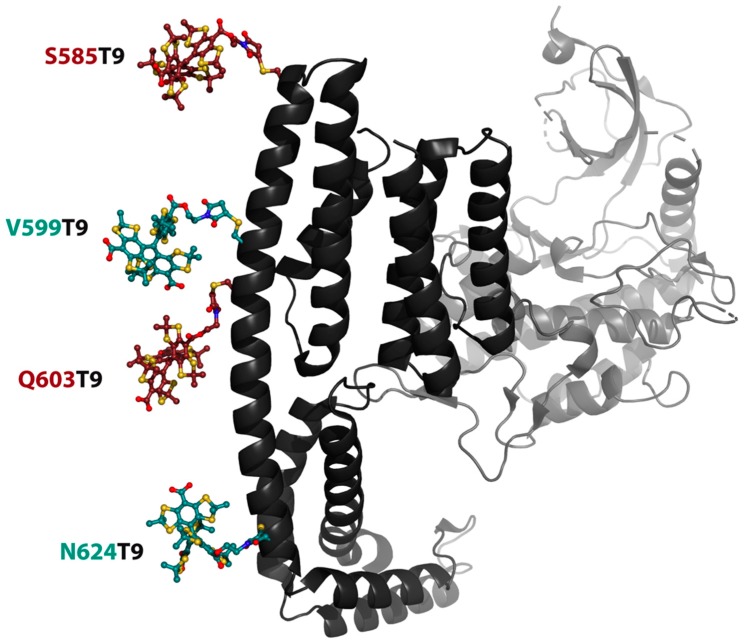
PyMOL cartoon representation of the GDI (front, black) and kinase domain (back, grey) of YopO (PDB-ID: 4ci6). The labeling positions on the GDI domain are indicated by color coded trityl pairs for the two studied mutants YopO S585C/Q603C (red) and YopO V559C/N624C (mint). For clarity reasons, only one conformer of **T9** is displayed for each labeling site.

Based on the findings made above, a labeling protocol was derived and then applied to the double cysteine YopO mutants S585C/Q603C and V559C/N624C. The introduced cysteine residues are located on the YopO GDI domain helix α14 [68] (Figure 5) whose rather rigid structure serves as a distance ruler that separates the labeling sites by five (S585C/Q603C) and seven (V599C/N624C) helix turns. According to the in silico predictions calculated with mtsslWizard [75], the expected mean distances between the trityl conformer clouds are 3.4 nm and 4.3 nm for YopO S585C/Q603C (red) and YopO V599C/N624C (mint), respectively.

**Figure 6:**
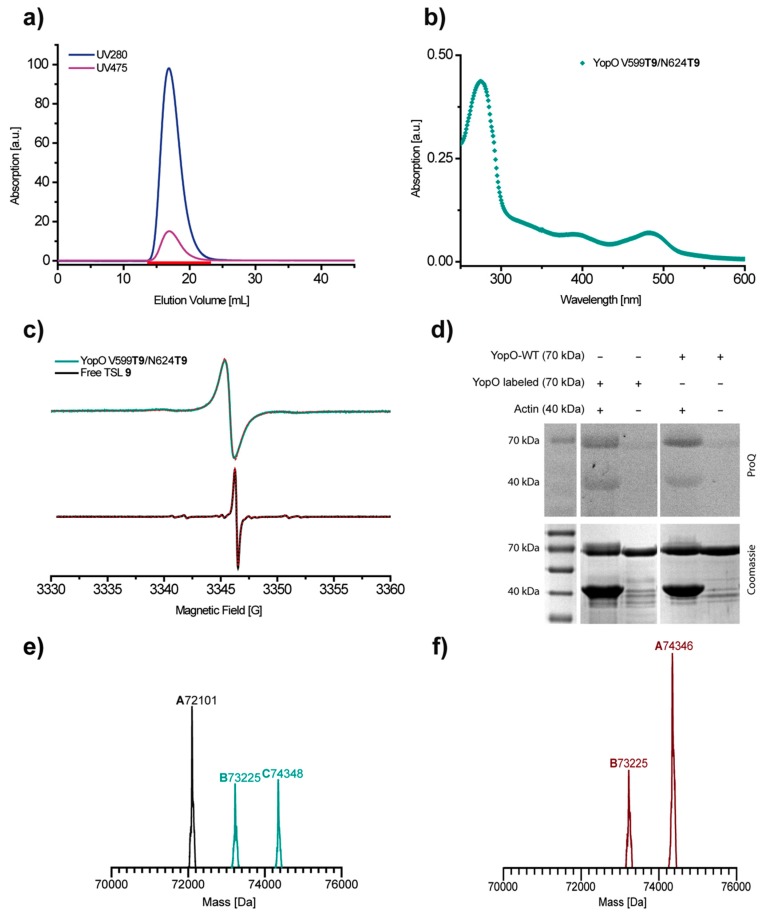
The labeling of YopO V599C/N624C with **9**. The analytics after the separation of the free label via PD-10 column. (**a**) HiPrep 26/10 size exclusion chromatogram of the labeled protein showing the trityl specific absorption at ~475 nm (red) and the absorption at 280 nm (blue). (**b**) UV/Vis spectrum of the labeled protein. The absorption maxima at ~475 nm and at ~280 nm are calculated to represent a concentration ratio of **9** to YopO of 1.8/1, i.e., 90% labeling efficiency. (**c**) The room temperature X-band *cw* EPR spectrum of the labeled protein YopO V599**T9**/N624**T9** (mint) as compared to the free label (black). The dashed red lines indicate spectral simulations obtained with EasySpin (Supplementary Materials, Section 8.2) [76]. EMXmicro acquisition parameters: modulation amplitude 0.15 G, microwave power 2.783 mW, time constant 20.48 ms, sweep time 42.04 s, resolution 67 pts/G. (**d**) The phosphorylation assay of labeled YopO V599**T9**/N624**T9** in comparison to YopO-WT. The phosphorylation is detected using ProQ Diamond stain and subsequent Coomassie staining. (**e**) ESI(+)-MS of the intact protein YopO V599**T9**/N624**T9**. The doubly labeled protein calculated: 74,347.6 Da, found: 74,348 Da (peak **C**). (**f**) ESI(+)-MS of the intact protein YopO S585**T9**/Q603**T9**. The doubly labeled protein calculated: 74,345.6 Da, found: 74,346 Da (peak **A**).

After incubation with **9**, purification and concentrating, the resulting protein solutions were subjected to analytical size exclusion chromatography, UV/Vis spectroscopy, *cw* EPR spin count experiments and ESI(+) mass spectrometry (MS). In addition, the functional and thus structural integrity of the labeled protein was checked using an assay to detect the autophosphorylation capability of YopO in the presence of actin [68]. Exemplarily, the assessment results for the doubly labeled YopO mutant V599**T9**/N624T**9** are displayed in Figure 6 (For the S585**T9**/Q603**T9** data set see Figure S8).

The size exclusion elugram (Figure 6a) shows that the trityl specific absorbance at ~475 nm is only detected in conjunction with the protein absorption band at ~280 nm. This finding rules out the presence of trityl aggregates and indicates the successful separation of free trityl. A trityl-protein ratio of 1.8/1 and thus a labeling efficiency of 90% was calculated from the UV/Vis absorbances at ~280 and ~475 nm (Figure 6b, for calculation see SI Section 4.2). The *cw* EPR spectrum of YopO V599**T9**/N624T**9** (mint trace in Figure 6c) is broadened as compared to free **9** (black trace in Figure 6c) but neither broader features indicative of aggregation nor narrow features indicative of free **9** are visible. As the observed line broadening is not accompanied by additional features and is straightforwardly simulated as a consequence of the trityl immobilization at the protein surface, the *cw* EPR spectrum corroborates the successful separation of the free label. Additionally, the *cw* EPR spin count reports a spin concentration of 87 µM at a protein concentration of 50 µM (Supplementary Materials, Section 8.1). Thus, the labeling efficiency as determined by UV/Vis (90%) matches within error the one determined by EPR (87%), which is much better than the 36% obtained previously for label **4**. The functional and thus structural integrity of the labeled YopO mutant is validated by a phosphorylation assay (Figure 6d). In accordance with the reported kinase activity of unlabeled YopO [67,68], both trityl-labeled mutants are phosphorylated in the presence but not in the absence of actin.

Finally, ESI(+) (Figure 6e) and MALDI(+) mass spectra (Figure S15a) of the protein sample reports the expected mass of 74348 Da (calculated: 74347.6 Da) for the doubly trityl-labeled sample (Figure 6e). In contrast to earlier reports [65], no threefold-labeled protein was detected, which points to the successful suppression of lysine labeling due to the chosen pH value of the labeling incubation. The additional mass peaks at 73225 Da and 72101 Da correspond to singly and unlabeled YopO, respectively. However, their intensities strongly vary for both YopO mutants and for the type of mass spectrometry used (Supplementary Materials, Section 5). This indicates that the non- and mono-labeled species are formed either during the mass spectrometry measurement and/or the preparation procedure for the mass spectrometry samples, which requires acidic conditions (trifluoracetic acid) and leads to the detachment of the label via retro-Michael reaction [77]. Several attempts to avoid the acidic MS-sample preparation failed. Consequently, the achieved labeling efficiencies are not reflected in the obtained MS spectra.

### 2.4. PDS Measurements

**Figure 7:**
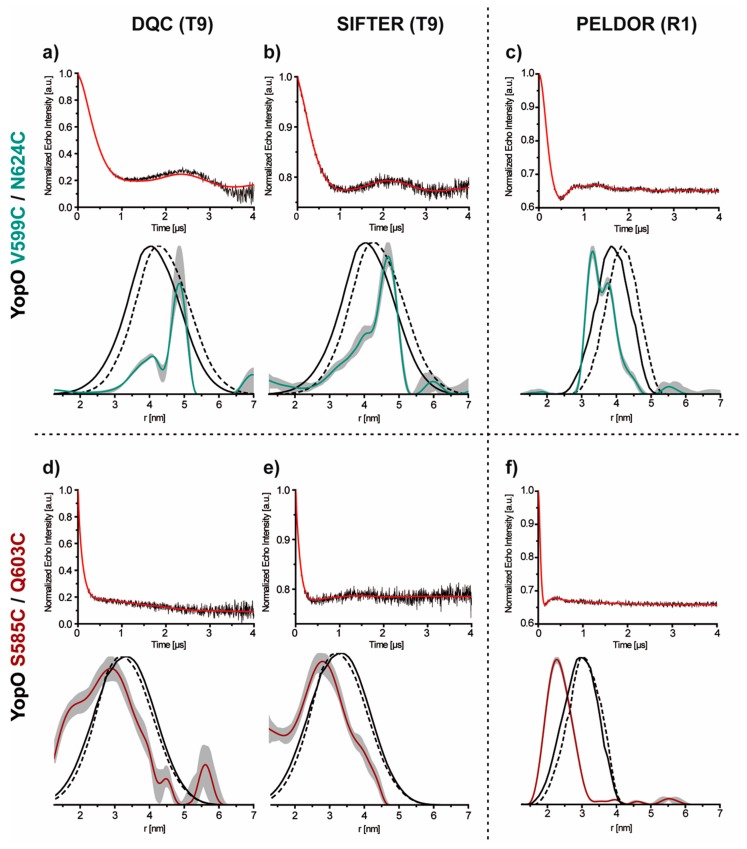
The PDS data of YopO V599C/N624C and S585C/Q603C. On the left, the background corrected DQC (**a**,**d**) and SIFTER (**b**,**e**) time traces (top) and their corresponding distance distributions (bottom) for YopO V599**T9**/N624**T9** and S585**T9**/Q603**T9**. On the right, the PELDOR (**c**,**f**) time traces (top) and the corresponding distance distributions (below) for YopO V599**R1**/N624**R1** (mint) and S585**R1**/Q603**R1** (red). In each case, the background corrected PDS time traces are shown in black, the corresponding fits from Tikhonov regularization in red. The distance distributions are shown in the respective color code while shaded grey areas indicate the error margins as obtained by the validation routine of DeerAnalysis [78]. The in silico predictions of the distance distributions were generated with mtsslWizard [75] and overlaid as dashed (PDB-ID 4ci6) and solid (PDB-ID 2h7o) black lines.

The doubly trityl labeled protein samples V599**T9**/N624**T9** and S585**T9**/Q603**T9** were measured with DQC and SIFTER [41], while the MTSSL labeled reference samples V599**R1**/N624**R1** and S585**R1**/Q603**R1**, were measured with PELDOR. The choice of the different pulse sequences is based on the different spectral width of the labels [2,6,9,11]. Figure 7 shows the background corrected time traces and the corresponding distance distributions (Figures S25 and S27, original time traces).

For the nitroxide- and trityl-labeled YopO mutant V599C/N624C, each time trace shows oscillations (Figure 7a–c, top) while mutant S585C/Q603C shows only strongly modulation damped time traces for both label types (Figure 7d–f, top). Regarding the modulation depths, both trityl-labeled mutants have modulation depths of 20–25% in the SIFTER [9,40,79] and of more than 80% in the DQC experiments. The differences in the modulation depth are attributed to the specifics of the two pulse sequences, especially the highly effective 64 step phase cycle in the case of DQC [7,80]. Nevertheless, the obtained modulation depth in DQC parallels those found for quantitatively labeled oligonucleotide samples [81] and model compounds [39,79] reflecting the high labeling efficiency and sample purity achieved here. The previously reported DQC modulation depths on trityl labeled proteins varied between 20–50% [43,65].

The signal-to-noise-ratios (SNR) [40,79,82] show that the trityl-trityl DQC (8.9 min^−1/2^ and 7.0 min^−1/2^, Figure 7a,d, top) and SIFTER experiments (5.8 min^−1/2^ and 5.9 min^−1/2^, Figure 7b,e, top) are more sensitive than the corresponding trityl-trityl PELDOR experiment (1.4 min^−1/2^ and 1.1 min^−1/2^, Figure S26). However, at a modulation depth of 35%, the nitroxide-nitroxide PELDOR measurements gave similar SNRs (9.9 min^–1/2^ and 7.3 min^–1/2^, Figure 7c,f, top) as the trityl-trityl DQC and SIFTER experiments. Thus, the sensitivity advantage of the trityl based single frequency experiments over nitroxide-nitroxide PELDOR measurements [1,6,9,11] is lost here due to two reasons: (1) The fivefold longer shot repetition time of 15 ms for the trityl labeled samples as compared to 3 ms for the MTSSL labeled samples (both at 50 K, Figure S20a+c) [41]. (2) The phase memory time *T_m_* for the trityl radicals (1.3 µs) is by a factor of 3.5 shorter than for the nitroxide radicals (4.6 µs, both at 50 K, Figure S20b+d) [42].

The corresponding distance distributions are shown in Figure 7a–f and are compared to the in silico labeling results. The in silico labeling was done with mtsslWizard in combination with the crystal structures of a YopO_89–729_ complex with actin (PDB-ID 4ci6, dashed black line) and a homologous structure of the truncated (amino acids 434–732) actin-free YpkA GDI domain from *Yersinia pestis* (PDB-ID 2h7o, solid black line). In each case, the experimental distance distributions are a subset of the in silico derived distributions. However, the **R1**/PELDOR derived distance distributions show a large shift of the most probable distance of up to 1 nm. For the **T9**-labeled samples, this distance shift is also observed although less marked, probably due to the different linker length/flexibility. In addition, mutant V599**C**/N624**C** reveals at least bimodal distance distributions for both, **R1** and **T9**. Both effects, the distance shift and the bimodality, may either be caused by preferred label conformations [2,83,84,85] and/or by YopO conformers with different bending degrees of the labeled α14 helix. The latter would fit to recent observations that the structure of YopO in solution seems to partially deviate from the crystal structure [86]. In any case, the new trityl spin label reproduces the results obtained with MTSSL.

## 3. Conclusions

The trityl-based spin label **9** was successfully synthesized, and by careful control of the labeling conditions, a labeling protocol could be established which enabled the site-selective bioconjugation of **9** to cysteines in high yields and without aggregations. This enabled PDS measurements between two trityl labels on YopO with good quality. Although the sensitivity advantage of trityl labels over nitroxide labels is lost here because of a fast *T_m_* relaxation, combining nitroxide and trityl labels with different functional groups enables orthogonal spin labeling. It could also be shown that the labeling of proteins with the methanethiosulfonate derivatized trityl compound **6** is compromised by the formation of a disulfide bridged bistrityl compound, which could not be separated from the protein. In order to improve the applicability and versatility of trityl labels in the future, new labels should display increased water solubility, e.g., by functionalizing the trityl OX063 instead of the Finland trityl. In addition, the linker group between trityl core and bioreactive moiety should be shortened and/or made more rigid to narrow the PDS-derived distance distributions.

## 4. Materials and Methods

### 4.1. Synthesis of ***9***

Under an atmosphere of argon, compound **5** (98.0 mg, 98.0 μmol) was dissolved in dry tetrahydrofuran (6 mL) and dry triethylamine (68 μL, 490 μmol). The mixture was stirred at room temperature for 30 min and then cooled to 0 °C. Consecutively, N(2-hydroxyethyl)maleimide (13.8 mg, 98.0 μmol), 2-chloromethylpyridinium iodide (CMPI, 33.2 mg, 130 μmol) and 4-dimethylaminopyridine (5.50 mg, 45.0 μmol) were given into the reaction which thereupon was allowed to warm to room temperature and stirred overnight. After 18 h, the reaction was quenched with aqueous HCl (0.36 M, 20 mL), the phases were separated, the aqueous layer was extracted with dichloromethane (2 × 10 mL) and the combined organic layers were concentrated under reduced pressure. According to MALDI-(+)-MS (Figure S1), this crude product mixture was composed of Finland trityl substrate, the one-fold and the two-fold 2-hydroxyethylmaleimide ester products. The crude product was coated onto silica gel (*w*/*w* = 1/3), packed into a cartridge which was mounted on a reversed phase column (Buechi FlasPure EcoFlex C18, 20 g, Büchi, Essen, Germany) and eluted with an acetonitrile gradient (10–100%) in deionized water (Figure S2). The product was isolated (Figures S3–S5) as a brown solid in a yield of 21% (22.7 mg, 20.2 μmol). Figure S6 shows UV/Vis and *cw* EPR spectra of the isolated compound **9** in buffer solutions. Figure S7 displays *cw* EPR spectra of compound **9** dissolved in organic solvents and revealing a A(^1^H) hyperfine coupling constant of 0.28 MHz and a g_iso_-value of 2.0035.

### 4.2. Protocol for Labeling YopO with ***9***

The protein (YopO, 20 nM in 2.5 mL) is incubated in the labeling buffer (20 mM POi, pH 6.8, 50 mM NaCl) with a five-fold molar excess of TCEP for 1.5 h at 4 °C in order to cleave disulfide bridged protein dimers. The remaining TCEP was removed using a PD-10 desalting column and the labeling reaction is set up immediately afterwards.

To the collected 3.5 mL protein solution is added a 5-fold molar excess per cystein of **9** (dissolved in 2.5 mL labeling buffer) resulting in a total volume of 6 mL containing 3.3 μM protein and 33 μM of **9**. The solution is incubated for 16 h at 4 °C. The free label excess was removed by loading fractions of 2 mL of the labeling solution onto a PD-10 size exclusion column (GE healthcare) followed by 500 μL of the labeling buffer and then eluting with 3.5 mL the labeling buffer. The total load of trityl on the PD10 should not exceed 70 nmol for maximum separation performance. The protein fraction was concentrated to 2.5 mL using a centrifugal concentrator (Vivaspin 6/10k MWCO, Sartorius, Goettingen, Germany).

### 4.3. UV-Vis Setup

For all UV/Vis experiments, 700 μL of the respective sample were loaded into a 0.7 mL Rotilabo^®^-precision quartz glass cuvette (Roth, Karlsruhe, Germany) and UV/Vis spectra were recorded from 600 to 200 nm at a rate of 0.3 s nm^−1^ using a Cary 100 UV-Vis (Agilent Technologies, Santa Clara, CA, USA).

### 4.4. Mass Spectrometry (MS) Setup

The ESI(+)-MS spectra were recorded on a LTQ Orbitrap Discovery spectrometer (Thermo Scientific, Waltham, MA, USA) while MALDI-MS spectra were obtained using an ultrafleXtreme TOF/TOF spectrometer (Bruker Daltonik, Bremen, Germany).

### 4.5. EPR Sample Preparation

The sample preparation for YopO labeled with **9**: After the UV/Vis concentration determination, all YopO-**T9** samples were spun down in a centrifugal concentrator (Vivaspin 6/10k MWCO) to volumes below 300 μL, rebuffered with 10 mL deuterated PELDOR buffer (100 mM TES pH 7.5, 100 mM NaCl) and concentrated to a final protein concentration of ~50 μM.

The sample preparation for YopO labeled with MTSSL: All YopO-R1 samples were rebuffered with 8 mL PELDOR buffer (100 mM TES pH 7.5, 100 mM NaCl). The samples were further concentrated to a final protein concentration > 50 μM.

*cw* EPR: The samples were loaded into a 10 μL glass capillary (Disposable Capillaries, Hirschmann^®^ Laborgeräte, Eberstadt, Germany), sealed with super glue and placed in an X-band tube (O.D. 4 mm, Wilmad-LabGlass).

Pulsed EPR: The samples were diluted 1:1 in deuterated ethylene glycol, transferred into a Q-band EPR tube (O.D. 3 mm, Wilmad LabGlass, Vineland, NJ, USA) and flash-frozen in liquid nitrogen.

### 4.6. EPR Setup

The room temperature *cw* EPR measurements were performed at X-band frequencies (~9 GHz) either on a Bruker (Bruker BioSpin, Rheinstetten, Germany) EMXmicro spectrometer equipped with an ER 4122SHQ resonator or on a Bruker EMXnano spectrometer (Bruker BioSpin, Rheinstetten, Germany) as stated in the respective figure captions.

The pulsed EPR measurements were conducted at Q-band frequencies (33.7 GHz) on a Bruker (Bruker BioSpin, Rheinstetten, Germany) ELEXSYS E580 EPR spectrometer (equipped with an ER 5106QT-II resonator and a 150 W TWT-amplifier (Applied Systems Engineering, Fort Worth, TX, USA). All data was acquired using quadrature detection. The temperature was adjusted to the appropriate value (between 50 K and 80 K) using a CF935 helium gas-flow cryostat (Oxford Instruments, Abingdon, UK) in conjunction with an Oxford Instruments ITC 502 temperature controller.

More detailed description of all methods and procedures can be found in the Supporting Information.

## Supplementary Materials

The following are available online at. It contains additional figures and experiments, full materials and methods including detailed and complete experimental procedures. EPR, UV/Vis, chromatography equipment, chemical synthesis and biosynthesis, EPR and MS sample preparations.

## References

[1] Schiemann O., Prisner T.F. (2007). Long-range distance determinations in biomacromolecules by EPR spectroscopy. Q. Rev. Biophys..

[2] Jeschke G. (2012). DEER Distance Measurements on Proteins. Annu. Rev. Phys. Chem..

[3] Timmel C.R., Harmer J.R. (2014). Structural Information from Spin-Labels and Intrinsic Paramagnetic Centres in the Biosciences. Struct. Bond..

[4] Milov A.D., Salikhov K.M., Schirov M.D. (1981). Application of ELDOR in electron-spin echo for paramagnetic center space distribution in solids. Fiz. Tverd. Tela (Leningrad).

[5] Milov A., Ponomarev A., Tsvetkov Y. (1984). Electron-electron double resonance in electron spin echo: Model biradical systems and the sensitized photolysis of decalin. Chem. Phys. Lett..

[6] Saxena S., Freed J.H. (1996). Double quantum two-dimensional Fourier transform electron spin resonance: Distance measurements. Chem. Phys. Lett..

[7] Saxena S., Freed J.H. (1997). Theory of double quantum two-dimensional electron spin resonance with application to distance measurements. J. Chem. Phys..

[8] Borbat P.P., Freed J.H. (1999). Multiple-quantum ESR and distance measurements. Chem. Phys. Lett..

[9] Jeschke G., Pannier M., Godt A., Spiess H. (2000). Dipolar spectroscopy and spin alignment in electron paramagnetic resonance. Chem. Phys. Lett..

[10] Kulik L., Dzuba S., Grigoryev I., Tsvetkov Y. (2001). Electron dipole–dipole interaction in ESEEM of nitroxide biradicals. Chem. Phys. Lett..

[11] Milikisyants S., Scarpelli F., Finiguerra M.G., Ubbink M., Huber M. (2009). A pulsed EPR method to determine distances between paramagnetic centers with strong spectral anisotropy and radicals: The dead-time free RIDME sequence. J. Magn. Reson..

[12] Park S.-Y., Borbat P.P., Gonzalez-Bonet G., Bhatnagar J., Pollard A.M., Freed J.H., Bilwes A.M., Crane B.R. (2006). Reconstruction of the chemotaxis receptor–kinase assembly. Nat. Struct. Mol. Boil..

[13] Banham J.E., Timmel C.R., Abbott R.J.M., Lea S.M., Jeschke G. (2006). The Characterization of Weak Protein–Protein Interactions: Evidence from DEER for the Trimerization of a von Willebrand Factor a Domain in Solution. Angew. Chem. Int. Ed..

[14] Denysenkov V.P., Prisner T.F., Stubbe J., Bennati M. (2006). High-field pulsed electron–electron double resonance spectroscopy to determine the orientation of the tyrosyl radicals in ribonucleotide reductase. Proc. Natl. Acad. Sci. USA.

[15] Pilotas C., Ward R., Branigan E., Rasmussen A., Hagelueken G., Huang H., Black S.S., Booth I.R., Schiemann O., Naismith J.H. (2012). Conformational state of the MscS mechanoselective channel in solution revealed by pulsed electron-electron double resonance (PELDOR) spectroscopy. Proc. Natl. Acad. Sci. USA.

[16] Herget M., Baldauf C., Schölz C., Parcej D., Wiesmüller K.-H., Tampé R., Abele R., Bordignon E. (2011). Conformation of peptides bound to the transporter associated with antigen processing (TAP). Proc. Natl. Acad. Sci. USA.

[17] Glaenzer J., Peter M.F., Thomas G.H., Hagelueken G. (2017). PELDOR Spectroscopy Reveals Two Defined States of a Sialic Acid TRAP Transporter SBP in Solution. Biophys. J..

[18] Abdullin D., Florin N., Hagelueken G., Schiemann O. (2015). EPR-Based Approach for the Localization of Paramagnetic Metal Ions in Biomolecules. Angew. Chem. Int. Ed..

[19] Schiemann O., Cekan P., Margraf D., Prisner T.F., Sigurdsson S.T. (2009). Relative Orientation of Rigid Nitroxides by PELDOR: Beyond Distance Measurements in Nucleic Acids. Angew. Chem..

[20] Marko A., Denysenkov V.P., Margraf D., Cekan P., Schiemann O., Sigurdsson S.T.H., Prisner T.F. (2011). Conformational Flexibility of DNA. J. Am. Chem. Soc..

[21] Stelzl L.S., Erlenbach N., Heinz M., Prisner T.F., Hummer G. (2017). Resolving the Conformational Dynamics of DNA with Ångstrom Resolution by Pulsed Electron–Electron Double Resonance and Molecular Dynamics. J. Am. Chem. Soc..

[22] Krstić I., Frolow O., Sezer D., Endeward B., Weigand J.E., Suess B., Engels J.W., Prisner T.F. (2010). PELDOR Spectroscopy Reveals Preorganization of the Neomycin-Responsive Riboswitch Tertiary Structure. J. Am. Chem. Soc..

[23] Kerzhner M., Matsuoka H., Wuebben C., Famulok M., Schiemann O. (2018). High-Yield Spin Labeling of Long RNAs for Electron Paramagnetic Resonance Spectroscopy. Biochemistry.

[24] Duss O., Michel E., Yulikov M., Schubert M., Jeschke G., Allain F.H.-T. (2014). Structural basis of the non-coding RNA RsmZ acting as a protein sponge. Nature.

[25] Wu Z., Feintuch A., Collauto A., Adams L.A., Aurelio L., Graham B., Otting G., Goldfarb D. (2017). Selective Distance Measurements Using Triple Spin Labeling with Gd3+, Mn2+, and a Nitroxide. J. Phys. Chem. Lett..

[26] Berliner L.J., Grunwald J., Hankovszky H., Hideg K. (1982). A novel reversible thiol-specific spin label: Papain active site labeling and inhibition. Anal. Biochem..

[27] Likhtenshtein G.I., Yamauchi J., Nakatsuji S., Smirnov A.I., Tamura R. (2008). Nitroxides: Applications in Chemistry, Biomedicine, and Materials Science.

[28] Kuzhelev A.A., Krumkacheva O.A., Shevelev G.Y., Yulikov M., Fedin M.V., Bagryanskaya E.G., Shevelev G.Y. (2018). Room-temperature distance measurements using RIDME and the orthogonal spin labels trityl/nitroxide. Phys. Chem. Chem. Phys..

[29] Azarkh M., Okle O., Eyring P., Dietrich D.R., Drescher M. (2011). Evaluation of spin labels for in-cell EPR by analysis of nitroxide reduction in cell extract of Xenopus laevis oocytes. J. Magn. Reson..

[30] Lawless M.J., Shimshi A., Cunningham T.F., Kinde M.N., Tang P., Saxena S. (2017). Analysis of Nitroxide-Based Distance Measurements in Cell Extracts and in Cells by Pulsed ESR Spectroscopy. ChemPhysChem.

[31] Karthikeyan G., Bonucci A., Casano G., Gerbaud G., Abel S., Thomé V., Kodjabachian L., Magalon A., Guigliarelli B., Belle V. (2018). A Bioresistant Nitroxide Spin Label for In-Cell EPR Spectroscopy: In Vitro and In Oocytes Protein Structural Dynamics Studies. Angew. Chem. Int. Ed..

[32] Yang Y., Yang F., Gong Y.-J., Bahrenberg T., Feintuch A., Su X.-C., Goldfarb D. (2018). High Sensitivity In-Cell EPR Distance Measurements on Proteins Using an Optimized Gd(III) Spin Label. J. Phys. Chem. Lett..

[33] Jassoy J.J., Berndhaeuser A., Duthie F., Kuehn S.P., Hagelueken G., Schiemann O. (2017). Versatile Trityl Spin Labels for Nanometer Distance Measurements on Biomolecules In Vitro and within Cells. Angew. Chem. Int. Ed..

[34] Jagtap A.P., Krstic I., Kunjir N.C., Hänsel R., Prisner T.F., Sigurdsson S.T.H. (2015). Sterically shielded spin labels for in-cell EPR spectroscopy: Analysis of stability in reducing environment. Free Radical Res..

[35] Yang Z., Bridges M.D., López C.J., Rogozhnikova O.Y., Trukhin D.V., Brooks E.K., Tormyshev V., Halpern H.J., Hubbell W.L. (2016). A Triarylmethyl Spin Label for Long-Range Distance Measurement at Physiological Temperatures Using T1 Relaxation Enhancement. J. Magn. Reson..

[36] Joseph B., Tormyshev V.M., Rogozhnikova O.Y., Akhmetzyanov D., Bagryanskaya E.G., Prisner T.F. (2016). Selective High-Resolution Detection of Membrane Protein-Ligand Interaction in Native Membranes Using Trityl-Nitroxide PELDOR. Angew. Chem..

[37] Gmeiner C., Klose D., Mileo E., Belle V., Marque S.R.A., Dorn G., Allain F.H.T., Guigliarelli B., Jeschke G., Yulikov M. (2017). Orthogonal Tyrosine and Cysteine Site-Directed Spin Labeling for Dipolar Pulse EPR Spectroscopy on Proteins. J. Phys. Chem. Lett..

[38] Reginsson G.W., Kunjir N.C., Sigurdsson S.T., Schiemann O. (2012). Trityl Radicals: Spin Labels for Nanometer-Distance Measurements. Chem. A Eur. J..

[39] Kunjir N.C., Reginsson G.W., Schiemann O., Sigurdsson S.T. (2013). Measurements of short distances between trityl spin labels with cw EPR, DQC and PELDOR. Phys. Chem. Chem. Phys..

[40] Akhmetzyanov D., Schöps P., Kunjir N.C., Sigurdsson S.T., Marko A., Prisner T.F. (2015). Pulsed EPR dipolar spectroscopy at Q- and G-band on a trityl biradical. Phys. Chem. Chem. Phys..

[41] Owenius R., Eaton G.R., Eaton S.S. (2005). Frequency (250 MHz to 9.2 GHz) and viscosity dependence of electron spin relaxation of triarylmethyl radicals at room temperature. J. Magn. Reson..

[42] Kuzhelev A.A., Trukhin D.V., Krumkacheva O.A., Strizhakov R.K., Rogozhnikova O.Y., Troitskaya T.I., Fedin M.V., Tormyshev V.M., Bagryanskaya E.G. (2015). Room—Temperature Electron Spin Relaxation of Triarylmethyl Radicals at the X- and Q-Bands. J. Phys. Chem. B.

[43] Yang Z., Liu Y., Borbat P., Zweier J.L., Freed J.H., Hubbell W.L. (2012). Pulsed ESR dipolar spectroscopy for distance measurements in immobilized spin labeled proteins in liquid solution. J. Am. Chem. Soc..

[44] Shevelev G.Y., Krumkacheva O.A., Lomzov A.A., Kuzhelev A.A., Rogozhnikova O.Y., Trukhin D.V., Troitskaya T.I., Tormyshev V.M., Fedin M.V., Pyshnyi D.V. (2014). Physiological-Temperature Distance Measurement in Nucleic Acid using Triarylmethyl-Based Spin Labels and Pulsed Dipolar EPR Spectroscopy. J. Am. Chem. Soc..

[45] Krumkacheva O., Bagryanskaya E. (2017). EPR-based distance measurements at ambient temperature. J. Magn. Reson..

[46] Andersson S., Rydbeck A., Mahno R.S. (1998). Free Radicals. US Patent.

[47] Reddy T.J., Iwama T., Halpern H.J., Rawal V.H. (2002). General Synthesis of Persistent Trityl Radicals for EPR Imaging of Biological Systems. J. Org. Chem..

[48] Dhimitruka I., Velayutham M., Bobko A.A., Khramtsov V.V., Villamena F.A., Hadad C.M., Zweier J.L. (2007). Large Scale Synthesis of a Persistent Trityl Radical for Use in Biomedical EPR Applications and Imaging. Bioorganic Med. Chem. Lett..

[49] Rogozhnikova O.Y., Vasiliev V.G., Troitskaya T.I., Trukhin D.V., Mikhalina T.V., Halpern H.J., Tormyshev V.M. (2013). Generation of Trityl Radicals by Nucleophilic Quenching of Tris(2,3,5,6-tetrathiaaryl)methyl Cations and Practical and Convenient Large-Scale Synthesis of Persistent Tris(4-carboxy-2,3,5,6-tetrathiaaryl)methyl Radical. Eur. J. Org. Chem..

[50] Hintz H., Vanas A., Klose D., Jeschke G., Godt A. (2019). Trityl Radicals with a Combination of the Orthogonal Functional Groups Ethyne and Carboxyl: Synthesis without a Statistical Step and EPR Characterization. J. Org. Chem..

[51] Liu Y., Villamena F.A., Sun J., Xu Y., Dhimitruka I., Zweier J.L. (2008). Synthesis and Characterization of Ester-Derivatized Tetrathiaarylmethyl Radicals as Intracellular Oxygen Probes. J. Org. Chem..

[52] Dhimitruka I., Bobko A.A., Hadad C.M., Zweier J.L., Khramtsov V.V. (2008). Synthesis and Characterization of Amino Derivatives of Persistent Trityl Radicals as Dual Function pH and Oxygen Paramagnetic Probes. J. Am. Chem. Soc..

[53] Decroos C., Prangé T., Mansuy D., Boucher J.-L., Li Y. (2011). Unprecedented ipso aromatic nucleophilic substitution upon oxidative decarboxylation of tris(pcarboxyltetrathiaaryl) methyl (TAM) radicals: A new access to diversely substituted TAM radicals. Chem. Commun..

[54] Driesschaert B., Marchand V., Leveque P., Gallez B., Marchand-Brynaert J. (2012). A phosphonated triarylmethyl radical as a probe for measurement of pH by EPR. Chem. Commun..

[55] Tormyshev V.M., Rogozhnikova O.Y., Bowman M.K., Trukhin D.V., Troitskaya T.I., Vasiliev V.G., Shundrin L.A., Halpern H.J. (2014). Preparation of Diversely Substituted Triarylmethyl Radicals by the Quenching of Tris(2,3,5,6-tetrathiaaryl)methyl Cations with C-, N-, P-, and S-Nucleophiles. Eur. J. Org. Chem..

[56] Driesschaert B., Bobko A.A., Khramtsov V.V., Zweier J.L. (2017). Nitro-Triarylmethyl Radical as Dual Oxygen and Superoxide Probe. Cell Biochem. Biophys..

[57] Tan X., Tao S., Liu W., Rockenbauer A., Villamena F.A., Zweier J.L., Song Y., Liu Y. (2018). Synthesis and Characterization of the Perthiatriarylmethyl Radical and Its Dendritic Derivatives with High Sensitivity and Selectivity to Superoxide Radical. Chem. A Eur. J..

[58] Fleck N., Hett T., Brode J., Meyer A., Richert S., Schiemann O. (2019). C–C Cross Coupling of Trityl Radicals: Spin Density Delocalization, Exchange Coupling, and a Spin Label. J. Org. Chem..

[59] Qu Y., Li Y., Tan X., Zhai W., Han G., Hou J., Liu G., Song Y., Liu Y. (2019). Synthesis and Characterization of Hydrophilic Trityl Radical TFO for Biomedical and Biophysical Applications. Chem. A Eur. J..

[60] Frank J., Elewa M., Said M.M., El Shihawy H.A., El-Sadek M., Muller D., Meister A., Hause G., Drescher S., Metz H. (2015). Synthesis, Characterization, and Nanoencapsulation of Tetrathiatriarylmethyl and Tetrachlorotriarylmethyl (Trityl) Radical Derivatives—A Study To Advance Their Applicability as in Vivo EPR Oxygen Sensors. J. Org. Chem..

[61] Marchand V., Levêque P., Driesschaert B., Marchand-Brynaert J., Gallez B., Marchand-Brynaert J. (2016). In vivo EPR extracellular pH-metry in tumors using a triphosphonated trityl radical. Magn. Reson. Med..

[62] Khramtsov V.V., Bobko A.A., Tseytlin M., Driesschaert B. (2017). Exchange Phenomena in the Electron Paramagnetic Resonance Spectra of the Nitroxyl and Trityl Radicals: Multifunctional Spectroscopy and Imaging of Local Chemical Microenvironment. Anal. Chem..

[63] Kishimoto S., Krishna M.C., Khramtsov V.V., Utsumi H., Lurie D.J. (2018). In Vivo Application of Proton-Electron Double-Resonance Imaging. Antioxid. Redox Signal..

[64] Peman A., Vilaseca M., Vitalla F.L., Van Doorslaer S., Marin-Montesinos I., Paniagua J.C., Pons M. (2016). Paramagnetic spherical nanoparticles by the self-assembly of persistent trityl radicals. Phys. Chem. Chem. Phys..

[65] Giannoulis A., Yang Y., Gong Y.-J., Tan X., Feintuch A., Carmieli R., Bahrenberg T., Liu Y., Su X.-C., Goldfarb D. (2019). DEER distance measurements on trityl/trityl and Gd(iii)/trityl labelled proteins. Phys. Chem. Chem. Phys..

[66] Song Y., Liu Y., Liu W., Villamena F.A., Zweier J.L. (2014). Characterization of the Binding of the Finland Trityl Radical with Bovine Serum Albumin. RSC Adv..

[67] Galyov E.E., Håkansson S., Forsberg A., Wolf-Watz H. (1993). A secreted protein kinase of Yersinia pseudotuberculosis is an indispensable virulence determinant. Nature.

[68] Lee W.L., Grimes J.M., Robinson R.C. (2015). Yersinia effector YopO uses actin as bait to phosphorylate proteins that regulate actin polymerization. Nat. Struct. Mol. Boil..

[69] Tan X., Chen L., Song Y., Rockenbauer A., Villamena F.A., Zweier J.L., Liu Y. (2017). Thiol-Dependent Reduction of the Triester and Triamide Derivatives of Finland Trityl Radical Triggers O2-Dependent Superoxide Production. Chem. Res. Toxicol..

[70] Jassoy J.J., Meyer A., Spicher S., Wuebben C., Schiemann O. (2018). Synthesis of Nanometer Sized Bis- and Tris-trityl Model Compounds with Different Extent of Spin–Spin Coupling. Molecules.

[71] Brewer C.F., Riehm J.P. (1967). Evidence for possible nonspecific reactions between N-ethylmaleimide and proteins. Anal. Biochem..

[72] Machida M., Machida M.I., Kanaoka Y. (1977). Hydrolysis of N-substituted maleimides: Stability of fluorescence thiol reagents in aqueous media. Chem. Pharm. Bull..

[73] Denes F., Pichowicz M., Povie G., Renaud P. (2014). Thiyl Radicals in Organic Synthesis. Chem. Rev..

[74] Nair D.P., Podgórski M., Chatani S., Gong T., Xi W., Fenoli C.R., Bowman C.N. (2014). ChemInform Abstract: The Thiol-Michael Addition Click Reaction: A Powerful and Widely Used Tool in Materials Chemistry. Chem. Mater..

[75] Hagelueken G., Ward R., Naismith J.H., Schiemann O. (2012). MtsslWizard: In Silico Spin-Labeling and Generation of Distance Distributions in PyMOL. Appl. Magn. Reson..

[76] Stoll S., Schweiger A. (2006). EasySpin, a comprehensive software package for spectral simulation and analysis in EPR. J. Magn. Reson..

[77] Baldwin A.D., Kiick K.L. (2011). Tunable degradation of maleimide-thiol adducts in reducing environments. Bioconjugate Chem..

[78] Jeschke G., Chechik V., Ionita P., Godt A., Zimmermann H., Banham J., Timmel C.R., Hilger D., Jung H. (2006). DeerAnalysis2006—A comprehensive software package for analyzing pulsed ELDOR data. Appl. Magn. Reson..

[79] Meyer A., Jassoy J.J., Spicher S., Berndhäuser A., Schiemann O. (2018). Performance of PELDOR, RIDME, SIFTER, and DQC in measuring distances in trityl based bi- and triradicals: Exchange coupling, pseudosecular coupling and multi-spin effects. Phys. Chem. Chem. Phys..

[80] Borbat P.P., Freed J.H., Goldfarb D., Stoll S. (2018). EPR Spectroscopy: Fundamentals and Methods.

[81] Shevelev G.Y., Gulyak E.L., Lomzov A.A., Kuzhelev A.A., Krumkacheva O.A., Kupryushkin M.S., Tormyshev V.M., Fedin M.V., Bagryanskaya E.G., Pyshnyi D.V. (2018). A Versatile Approach to Attachment of Triarylmethyl Labels to DNA for Nanoscale Structural EPR Studies at Physiological Temperatures. J. Phys. Chem. B.

[82] Bahrenberg T., Yang Y., Goldfarb D., Feintuch A. (2019). rDEER: A Modified DEER Sequence for Distance Measurements Using Shaped Pulses. Magnetochemistry.

[83] Hagelueken G., Ingledew W.J., Huang H., Petrovic-Stojanovska B., Whitfield C., ElMkami H., Schiemann O., Naismith J.H. (2009). PELDOR Spectroscopy Distance Fingerprinting of the Octameric Outer-Membrane Protein Wza fromEscherichia coli. Angew. Chem..

[84] Jeschke G. (2014). Interpretation of Dipolar EPR Data in Terms of Protein Structure. Struct. Bond..

[85] Abdullin D., Hagelueken G., Schiemann O. (2016). Determination of nitroxide spin label conformations via PELDOR and X-ray crystallography. Phys. Chem. Chem. Phys..

[86] Peter M.F., Tuukkanen A.T., Heubach C.A., Selsam A., Duthie F.G., Svergun D.I., Schiemann O., Hagelueken G. (2019). Studying Conformational Changes of the Yersinia Type-III-Secretion Effector YopO in Solution by Integrative Structural Biology. Structure.

