# Supplementary material for: Site Selective and Efficient Spin Labeling of Proteins with a Maleimide-Functionalized Trityl Radical for Pulsed Dipolar EPR Spectroscopy
Source: Molecules. 2019 Jul 27;24(15):2735. doi: 10.3390/molecules24152735 (PMC6696014; doi:10.3390/molecules24152735)
Supplement: Supplementary file 1 [file molecules-24-02735-s001.pdf]

Site Selective and Efficient Spin Labeling of Proteins with a Maleimide-Functionalized Trityl Radical for Pulsed Dipolar EPR Spectroscopy

*J. Jacques Jassoy‡, Caspar A. Heubach‡, Tobias Hett, Frédéric Bernhard, Florian R. Haege, Gregor Hagelueken, Olav Schiemann**

Institute of Physical and Theoretical Chemistry, Rheinische Friedrich-Wilhelms-University Bonn, Wegelerstr. 12, 53115 Bonn, Germany, email: schiemann@pc.uni-bonn.de, phone: +49(0)228732989, fax: +49(0)228732073

‡ These authors equally contributed to this work.

Supporting Information

Table of Contents

1. Synthesis and Characterization of **9** S3
2. Analytical Data for YopO S585**T9**/Q603**T9** and V599**T9**/N624**T9** S6
3. Activity Assay S7
4. UV/Vis Quantification Methods S7
4.1 Dilution series S7
4.2 Calibration Curve for **9** S8
4.3 Deconvolution of UV/Vis spectra S9
5. MS Analyses of Labeled YopO S10
5.1 MS Protein Sample Preparations S10
5.2 ESI(+)-MS on YopO-WT after labeling incubation with **9** S10
5.3 MALDI(+)-MS of YopO V599**T9**/N624**T9** and S585**T9**/Q603**T9** S11
6. Dimer Formation of **6** and Cleavage S11
7. MTSSL Labeling of YopO S12
8. EPR Measurements S13
8.1 Spin Count S13
8.2 Simulation of cw EPR Spectra S13
8.3 Relaxation Time Measurements S14
9. PDS Measurements S15
9.1 DQC S15
9.2 SIFTER S16
9.3 PELDOR S16
9.4 Original PDS Time Traces, Background Removal and Validation S17
9.5 Signal to Noise Ratio (SNR) determination S21
10. Protein Work S22
10.1 Mutagenesis S22
10.2 Expression and Purification S23
11. References S25

1. Synthesis and Characterization of 9

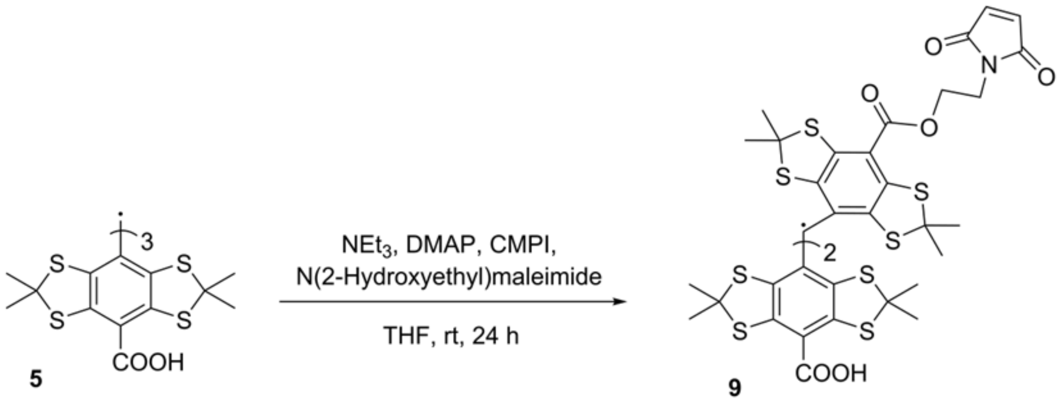

Scheme S1. Reaction leading to compound **9**. DMAP = dimethylaminopyridine, CMPI = 2-chloro-methylpyridinium iodide, THF = tetrahydrofuran.

MS (MALDI+) *m/z*: $[M]^{\bullet+}$ 1121.9 (100).

HRMS (ESI(+)) *m/z*: $[M]^{\bullet+}$ calcd for $[C_{46}H_{44}NO_8S_{12}]^{\bullet+}$: 1121.9710, found: 1121.9707.

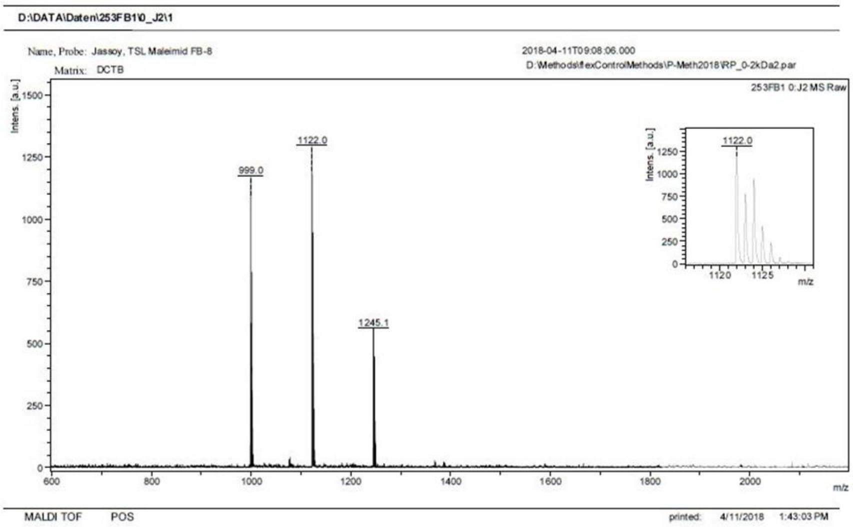

Figure S1. MALDI-(+)-MS of the crude product mixture after aqueous work-up. m/z 999.0 = Finland Trityl, m/z 1122.0 = [**9**], m/z = 1245.1 = [twofold ester product].

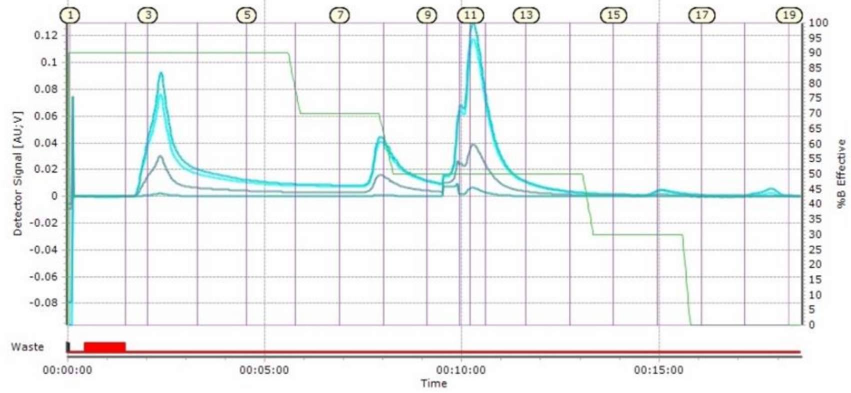

Figure S2. Elugram obtained during the reversed phase (C18) MPLC purification of the crude product mixture. A gradient of acetonitrile (10%-100%) in water was applied. Compound **9** was eluted in fractions 11 and 12.

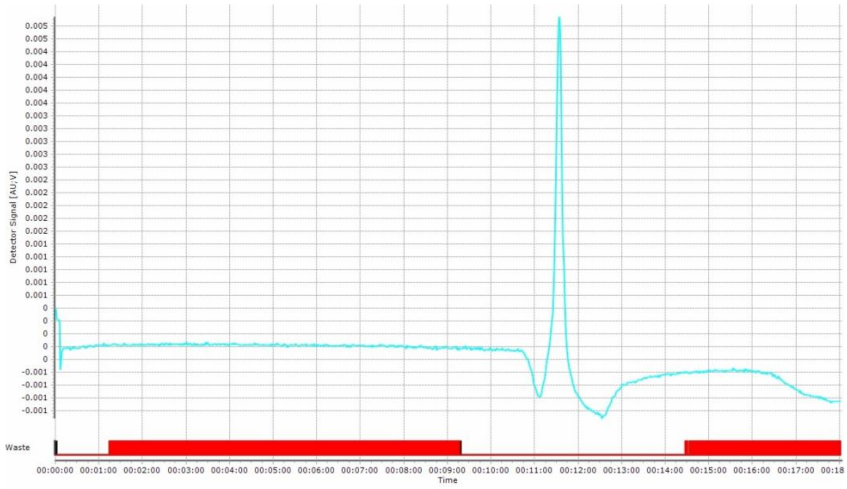

Figure S3. Reversed phase (C18) MPLC purity assessment elugram of **9**. A gradient of acetonitrile (10%-100%) in water was applied to elute a 1 mg sample of **9**.

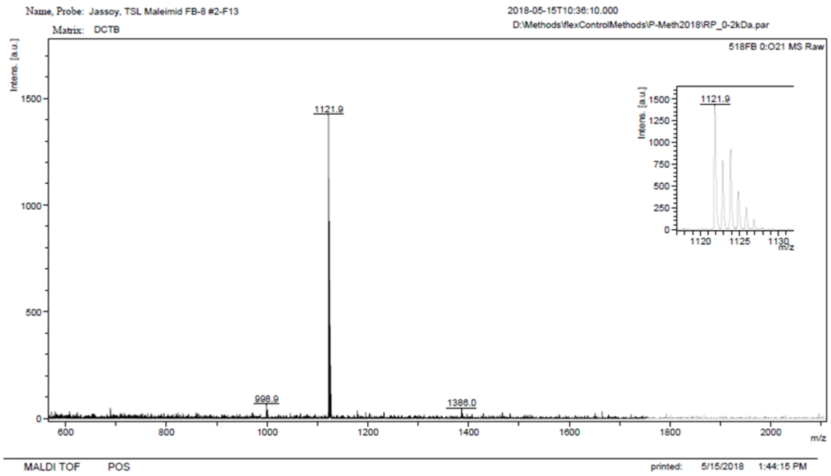

Figure S4. MALDI-(+)-MS of combined fractions 11 and 12 after MPLC purification. m/z: 999.9 (5) [Finland Trityl+H], 1121.9 (100) [**9**], 1380.0 (3) [unknown].

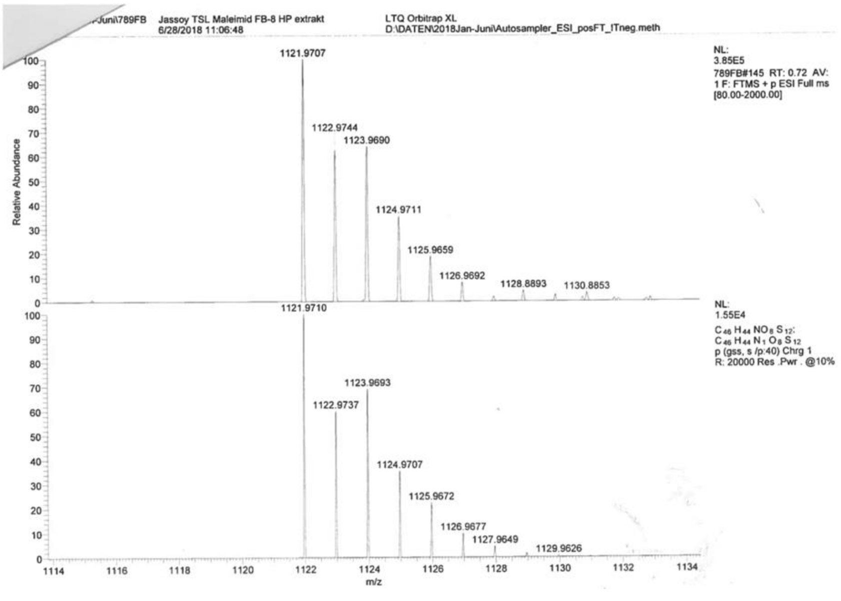

Figure S5. ESI-(+)-HRMS of **9**. Top panel: m/z = 1121.9707 found, bottom panel: m/z = 1121.9710 calculated.

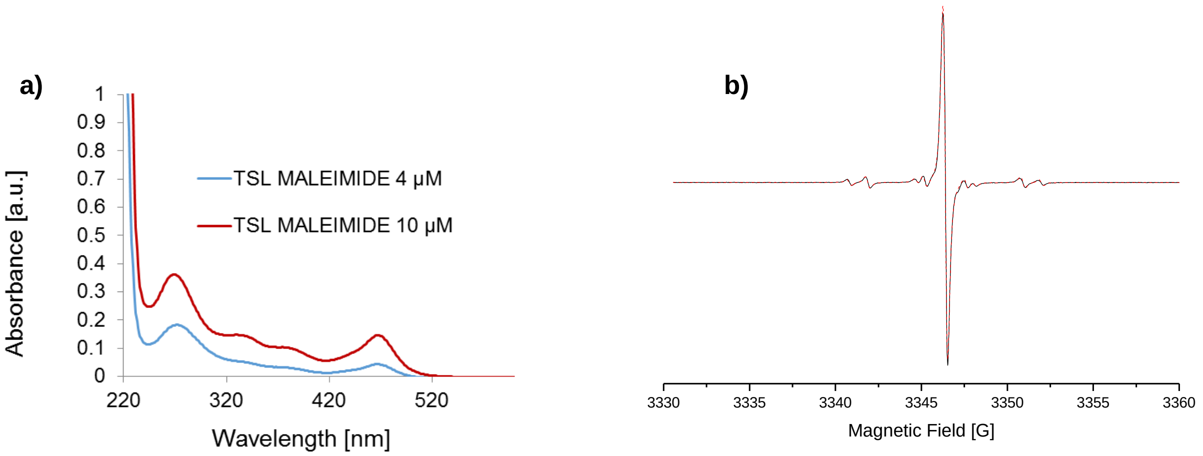

Figure S6. a) UV/Vis spectrum of **9** at 4 µM (blue) and at 10 µM (red) in TRIS buffer, pH 7.0. **b)** Room temperature *cw* EPR spectrum of **9** at 100 µM in 10 mM TES buffer (100 mM NaCl), pH 7.4 (black trace), superimposed with the corresponding EasySpin[3] simulation (red trace). EMXmicro, modulation amplitude: 0.150 G, microwave power: 2.756 mW, time constant: 20.48 ms, resolution: 67 pts/G. Simulation parameters are declared in Table S3, entry c.

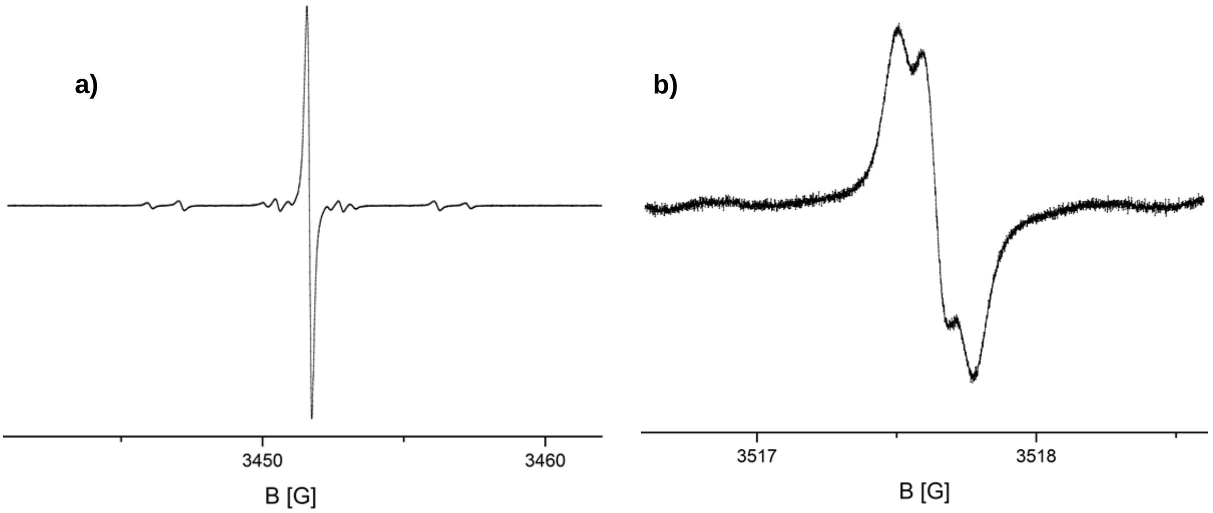

Figure S7. a) Room temperature *cw* EPR spectrum of **9** at 67 µM in degassed DMSO. EMXmicro, modulation amplitude: 0.008 G, microwave power: 794 µW, time constant: 5.12 ms, sweep time: 449.98 s, resolution: 250 pts/G, frequency: 9.637744 GHz, g-value = 1.9949637, ^{13}C coupling constants = 3.15 MHz, 12.68 MHz, 15.82 MHz. **b)** Room temperature *cw* EPR spectrum of TSL **9** at 67 µM in degassed DCM. EMXmicro, modulation amplitude: 0.030 G, microwave power: 929 µW, time constant: 10.24 ms, sweep time: 320 s, resolution: 2000 pts/G, frequency: 9.863920 GHz.

2. Analytical Data for YopO S585T9/Q603T9 and V599T9/N624T9

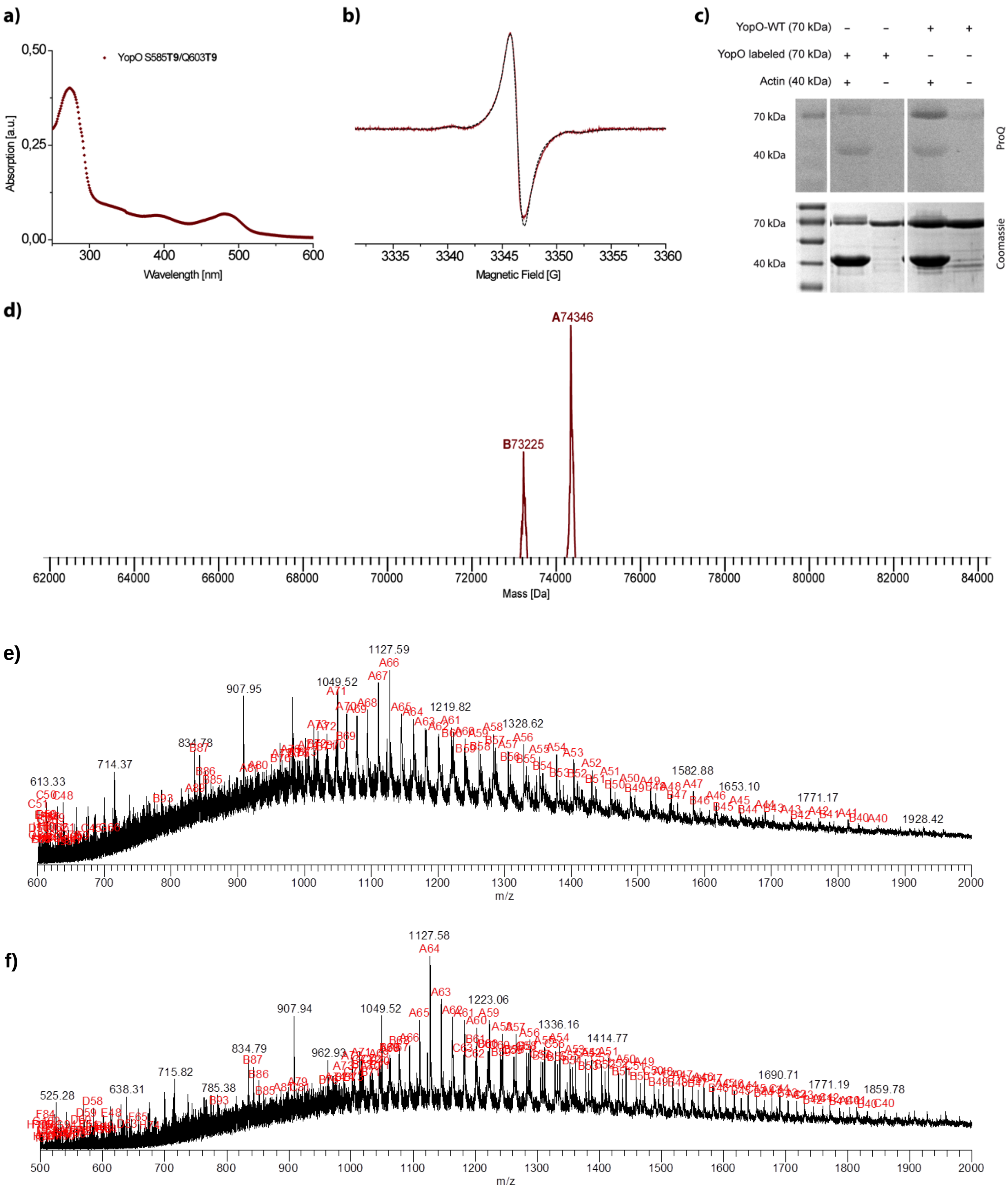

Figure S8. (a) UV/Vis absorption spectrum of YopO S585**T9**/Q603**T9**. Molar ratio of YopO to **9** yields quantitative labeling of both cysteines using equation (I) and (II) in SI section 4.2 (b) *cw* EPR spectrum recorded at an EMXmicro of YopO S585**T9**/Q603**T9** (~50 µM). Quantitative spin count via double integration against a 100 µM reference sample of free TSL **9** gave a relative spin concentration of 90 µM. A simulated spectrum is overlaid as a dashed black line. (c) Phosphorylation assay setup for YopO S585**T9**/Q603**T9** showing the catalytic activity of the protein. (d) Deconvoluted ESI(+)-MS of the sample. Doubly labeled protein calculated: 74345.6 Da, found: 74346 Da (Peak **A**); singly labeled protein calculated: 73222.6 Da, found: 73225 Da (Peak **B**). e) Raw ESI(+)-MS spectrum of YopO S585**T9**/Q603**T9**. f) Raw ESI(+)-MS spectrum of YopO V599**T9**/N624**T9** (supplement for deconvoluted ESI(+)-MS of maintext Figure 6e).

3. Activity Assay

2 µM YopO-WT and YopO labeled with **9**, respectively, were incubated in the presence of 6 µM G-actin in phosphorylation buffer (50 mM Tris•HCl pH 8.0, 10 mM $MgCl_2$, 1 mM ATP, 2 mM $MnCl_2$) for 30 min. at 37 °C. A sample without G-actin in the incubation mixture served as a negative control. The assay was quenched by the addition of 8x SDS buffer and subsequent boiling of the sample at 95 °C for 5 min. SDS-PAGE gels were first fixed in 45% MeOH, 10% AcOH (2 times, 30 min, 100 mL), washed with MilliQ water (3 times, 10 min, 100 mL) and then stained in the dark with Pro-Q Phosphoprotein Diamond Stain (Thermo Fisher Scientific) for 90 min. Excess staining solution was removed in 20% Acetonitrile, 50 mM NaOAc pH 4.0 (3 times, 30 min, 100 mL) and gels were washed with MilliQ water (2 times, 5 min, 100 mL). Gels were imaged at a UV table using a 590 nm longpass emission filter. Subsequently, the gels were stained in Coomassie for visualization of total protein.

4. UV/Vis Quantification Methods

4.1 Dilution series

The following figures (Figure S9–S11) summarize the UV/Vis absorption of **5**, **6** and **9** at different concentrations and pH values in phosphate buffer solutions (20 mM PO_i, 50 mM NaCl).

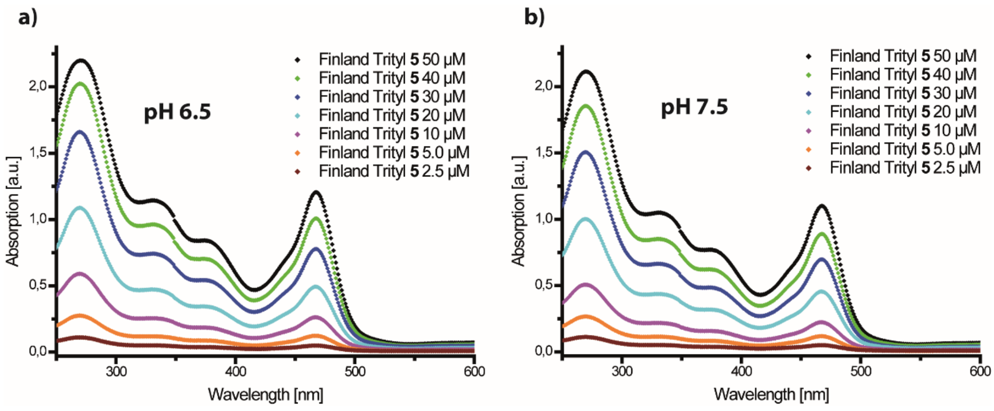

Figure S9. UV/Vis spectra of 2.5–50 µM **5** in PO_i buffer pH 6.5 (a) and pH 7.5 (b).

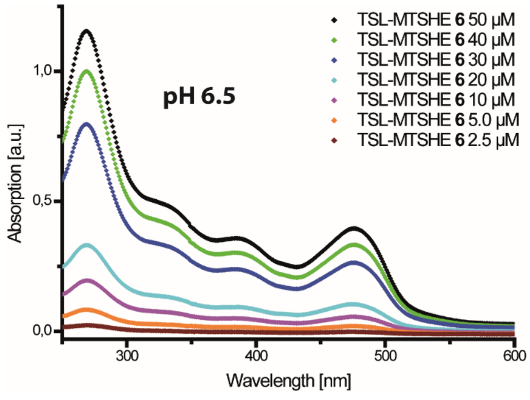

Figure S10. UV/Vis spectra of 2.5–50 µM **6** in PO_i buffer pH 6.5.

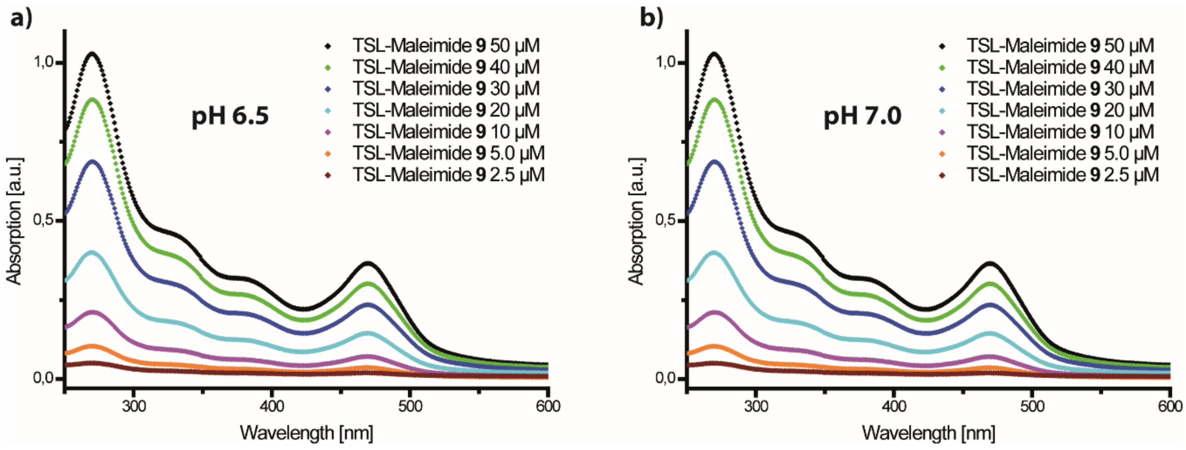

Figure S11. UV/Vis spectra of 2.5–50 µM **9** in POi buffer pH 6.5 (a) and pH 7.0 (b).

In the case of **5**, the absorbance of the trityl is slightly increased in an acidic buffer system (Figure S9a) as compared to alkaline conditions (Figure S9b). For **6**, the UV/Vis absorbance shows a strong decrease in the absorbance going from a 30 µM concentration to 20 µM (Figure S10). This may be related to the experimental findings in section 6 below. For **9**, the dilution series at pH 6.5 (Figure S11a) and pH 7.0 (Figure S11b) show no significant differences at a given concentration.

4.2 Calibration Curve for 9

For **9**, the concentration dependent absorbance at 467 nm and 280 nm is plotted in Figure S12 with the resulting linear equations displayed in Table S1.

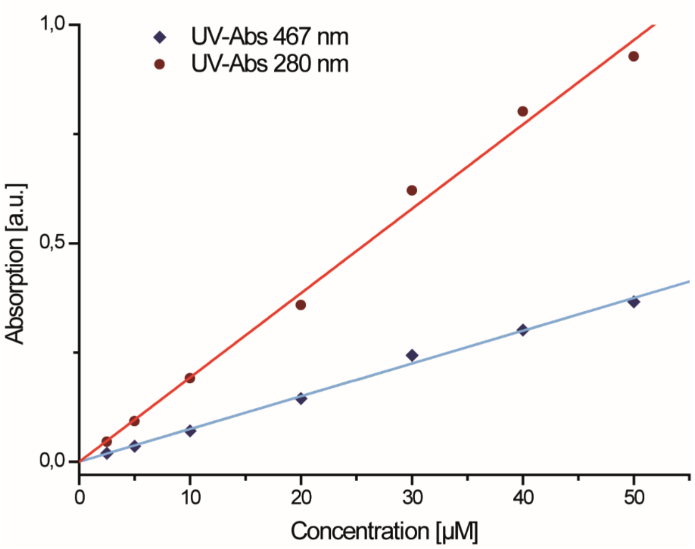

Figure S12. UV/Vis calibration curve of TSL **9** (in PO_i buffer pH 6.5) for the absorption at 467 nm and 280 nm. Linear regression parameters are summarized in Table S1.

Table S1. Linearized equations of the calibration curves shown in Figure S16.

Wavelength [nm]	Linear Equation
280	$(\mathbf{I})\ \mathrm{Abs}_{280} = 0.0193\frac{\mathrm{a.u.}}{\mu\mathrm{M}}\ [\mathrm{TSL}\ \mathbf{9}] - 1.725 \cdot 10^{-4}\ \mathrm{a.u.}$
467	$(\mathbf{II})\ \mathrm{Abs}_{467} = 0.0075\frac{\mathrm{a.u.}}{\mu\mathrm{M}}\ [\mathrm{TSL}\ \mathbf{9}] - 4.604 \cdot 10^{-4}\ \mathrm{a.u.}$

In order to evaluate the molar ratio between **9** and YopO in the labeling experiments, the absorbance peak of **9** at ~467 nm is used to determine the concentration of **9** in a labeled sample via equation (**II**). Knowing the concentration of **9**, the contribution of the label to the absorbance at ~280 nm can be estimated by means of the linear equation (**I**) and the remaining absorption is attributed to the protein fraction in the respective sample. Since a slight bathochromic shift of the local absorbance maximum at 467 nm of **9** and small spectral deviations after the labeling reactions were observed, concentrations were determined using the maximum absorbance peaks at ~467 nm and ~280 nm. For YopO, an extinction coefficient was obtained of $\varepsilon_{280} = 0.04939\frac{a.u.}{\mu M}$ using the web-based peptide parameter computing tool ProtParam.

4.3 Deconvolution of UV/Vis spectra

In order to evaluate the accuracy of the concentration determinations outlined above the following experiment was performed: UV/Vis spectra of an unlabeled 4.05 µM YopO solution and a 10 µM solution of **9** in labeling buffer (recorded at a Cary 100 UV-Vis) were fitted (Figure S13a) to the function

$$[\mathrm{Sample}] = (\mathrm{a} \cdot [\mathrm{YopO}] + (1 - \mathrm{a}) \cdot [\mathrm{TSL}\ \mathbf{9}]) \cdot \mathrm{b} + \mathrm{c}$$

where b denotes the scaling factor and c corresponds to an offset correction factor. These fits were then used to deconvolute the UV-vis spectra of labeled YopO mutants V599**T9**/N624**T9** (Figure S13b) and YopO S585**T9**/Q603**T9** (Figure S13c). Table S2 summarizes the quantification by both methods.

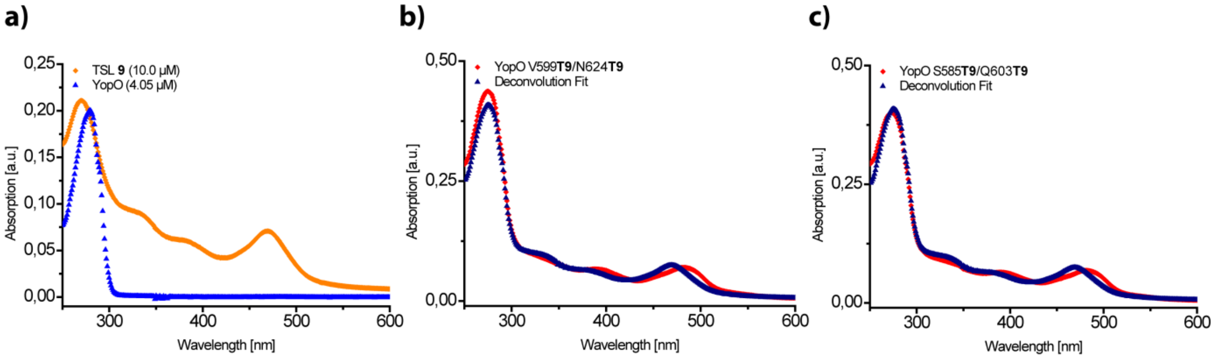

Figure S13. UV/Vis spectra of 4.05 µM YopO (blue) and 10 µM TSL **9** (red) in labeling buffer (a). Recorded UV/Vis spectra after the labeling reaction and excess label removal (marine) and the corresponding deconvolution fit (red) of YopO V599**T9**/N624**T9** (b) and YopO S585**T9**/Q603**T9** (c).

Table S2. Concentrations of YopO and **9** in the spectra of (Figure S13 b and c) determined using either the deconvolution or the maximum peak method.

Sample	Deconvolution	Maximum Peak
**(a) YopO V599C/N624C**	4.65 µM YopO<br>10.68 µM **9**	5.20 µM YopO<br>9.33 µM **9**
**(b) YopO S585C/Q603C**	3.90 µM YopO<br>10.94 µM **9**	4.56 µM YopO<br>9.13 µM **9**

Comparing the resulting concentrations of YopO and **9** using either the deconvolution or the maximum peak value method, the deconvolution method gives slightly lower concentrations of YopO and slightly higher concentrations of **9**. However, the deconvolution function is not able to correct for the bathochromic shift in the absorbance of **9**, leading to unsatisfactory fits in the region above 300 nm. Overall, both methods give sufficiently accurate results for the determination of the concentrations of YopO and **9**.

5. MS Analyses of Labeled YopO

5.1 MS Protein Sample Preparations

ESI(+)-MS: The protein solutions were desalted with an Amicon centricon by exchanging the buffer with a mixture of 0.1% formic acid and 20% acetonitrile in milliQ water. The exchange was performed adding 3x 500 µL to 50 µL of protein buffer solution. This solution was then injected into the ESI-MS instrument.

MALDI(+)-MS: The protein buffer solutions were mixed with matrix solution (2,5-dihydroxyacetophenon (DHAP) in ethanol with diammonium hydrogencitrate) and a 2% trifluoroacetic acid (TFA) solution in water. The resulting suspension was transferred to a stainless steel MALDI target and evaporated to dryness at room atmosphere conditions.

5.2 ESI(+)-MS on YopO-WT after labeling incubation with 9

In addition to UV/Vis and *cw* EPR (main text Figure 6) also the ESI(+)-MS spectra show only unlabeled YopO-WT (Figure S14).

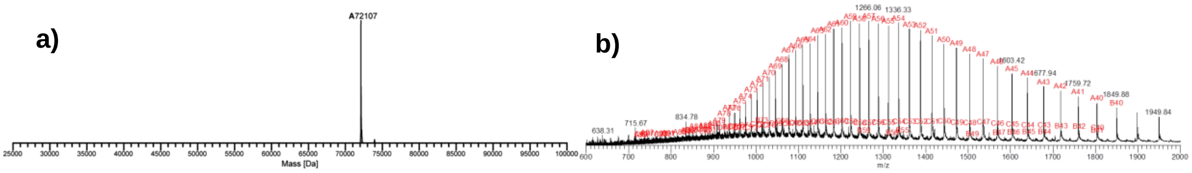

Figure S14. a) Deconvoluted ESI(+)-MS of 0-cysteine YopO-WT incubated with **9**, calculated: 72108 Da, found: 72107 Da. b) Raw ESI(+)-MS spectrum of YopO-WT incubated with **9**.

5.3 MALDI(+)-MS of YopO V599T9/N624T9 and S585T9/Q603T9

Both MALDI-MS analyses show masses of the respective non-, onefold- and twofold-labeled YopO mutants. This is inconsistent with the labeling degree distributions reported by ESI(+)-MS (Figure 6 of the main text). Also, *cw* EPR and UV/Vis report a high labeling efficiency, which is not reflected in these MS results. However, the MALDI as well as the ESI sample preparation required acidic conditions (2% trifluoroacetic acid for MALDI and 0.1% formic acid for ESI(+)), which promote partial label detachment via retro-Michael reactions before and during the MS measurements. Several attempts to skip the acidic sample preparation failed.

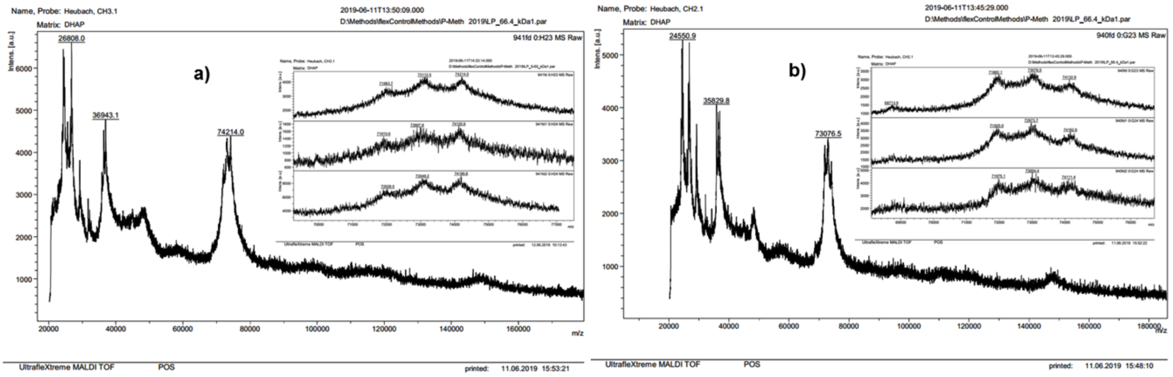

Figure S15. MALDI-MS of YopO V599**T9**/N624**T9** (a) and of YopO S585**T9**/Q603**T9** (b).

6. Dimer Formation of 6 and Cleavage

Two separated 25 µM solutions of **6** and **9** in labeling buffer (20 mM PO_i pH 6.8, 50 mM NaCl) were incubated for 16 h at 4 °C under the exclusion of light. The solutions were spun down to a final volume of 300 µL using VivaSpin 2/10k MWCO. From each solution a part was transferred into a 10 µL glass capillary and *cw* EPR spectra were recorded using a Bruker EMXnano spectrometer (Figure S16). Then the sample of **6** was irradiated with UV light (Figure S17).

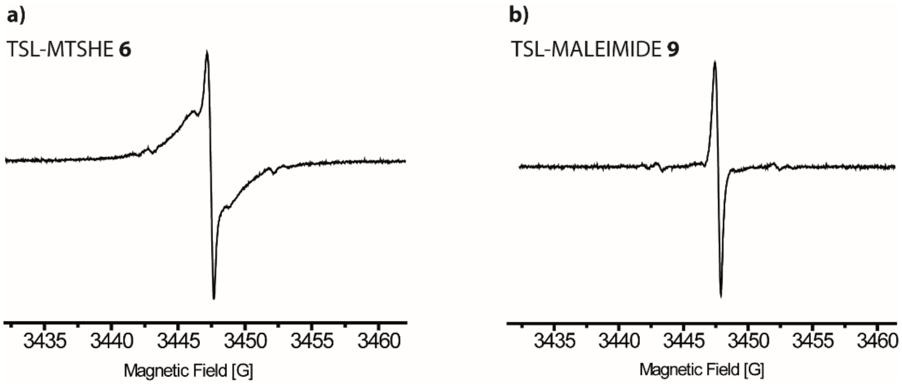

Figure S16. *cw* EPR spectrum of **6** (a) and **9** (b) after incubation of the trityls in PO_i buffer pH 6.8 for 16 h at 4 °C and subsequent centrifugation by means of a Vivaspin 2/10k MWCO centricon.

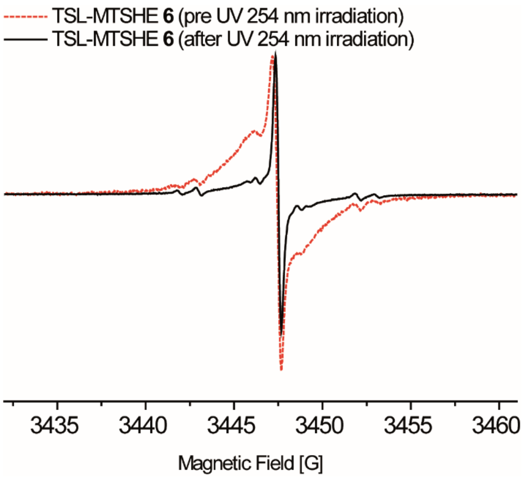

Figure S17. *cw* EPR spectra of **6** prior to (red, dashed) and after (black, solid) irradiation with UV light of 254 nm for 10 min.

The determined ratio of the double integrals before and after UV irradiation (Figure S17) is 1 **:** 0.92. Taking the error of the method into account (20%), this means that the number of spins does not change upon UV irradiation, which further supports the hypothesis of disulfide bridging between two molecules of **6** in prolonged incubations. In contrast to the case of **6**, the *cw* EPR of **9** does not display a similar line shape broadening (Figure S16b) after incubation, indicating that **9** remains unchanged under the labeling conditions.

7. MTSSL Labeling of YopO

For the MTSSL references, YopO V599C/N624C and YopO S585C/Q603C were incubated in 3 mM DTT for 30 min at room temperature. After reducing agent removal via PD10 size exclusion chromatography, the protein containing fraction was incubated with a 10-fold molar excess of MTSSL per cysteine in MTSSL labeling buffer (50 mM Tris•HCl pH 8.0, 50 mM NaCl) for 16 h at 4 °C. The excess free spin label was removed using a centrifugal concentrator (Vivaspin 2/10k MWCO).

8. EPR Measurements

8.1 Spin Count

The room temperature *cw* EPR spectra of both YopO mutants were used to derive the labeling efficiency in reference to the free TSL **9** buffer solution of known concentration (100 µM) (Figure S18).

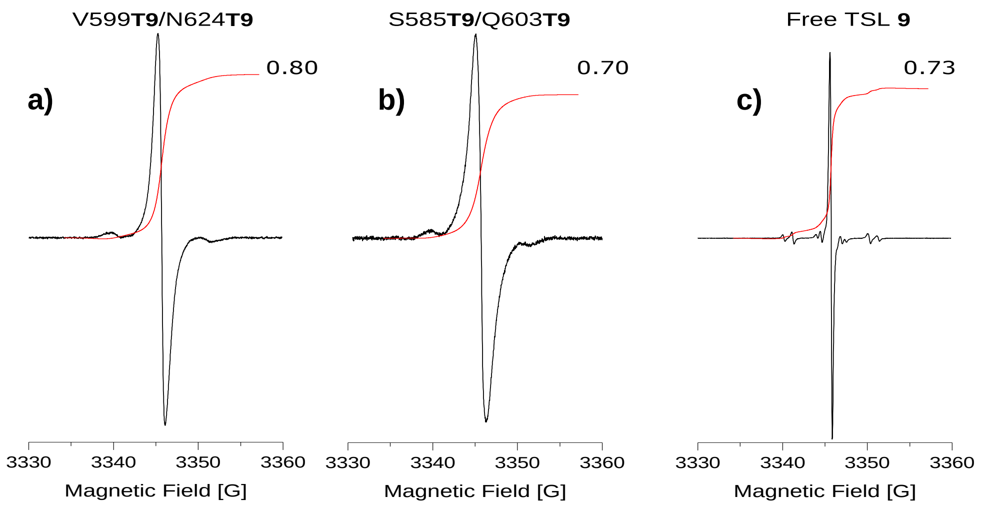

Figure S18. Room temperature *cw* EPR spectra obtained from YopO mutants (a) V599**T9**/N264**T9** (109% labeling efficiency) and (b) S585**T9**/Q603**T9** (95% labeling efficiency). The spin concentrations of the protein samples were determined in reference to the 100 µM **TSL 9** solution in buffer (c).

8.2 Simulation of cw EPR Spectra

The cw EPR spectra of YopO V599**T9**/N624**T9** (Table S3, entry a; Figure 6 in the main text) and S585**T9**/Q603**T9** (Table S3, entry b; Figure S8b) were simulated using the "chili" routine of EasySpin[1] taking into account g-anisotropy and a rotational correlation time τ. The hyperfine coupling tensor was assumed to be isotropic. The spectrum of free label **9** (Table S3, entry c; Figure S6b) was simulated using the "garlic" routine of EasySpin.[1] All simulation parameters are summarized in Table S3. The range of the fitted values is in accordance with the literature.[2,3] Assignments of hyperfine coupling constants to explicit ^{13}C nuclei in the case of **9** was done according to Bowman *et al*.[3]

Table S3. EasySpin simulation parameters.

Sample	Simulation Parameter
**(a) YopO V599C/N624C**	g = (2.0041, 2.0043, 2.0015)<br>A = 30.8 MHz<br>LWPP = (0, 0.035) mT<br>τ = 15 ns
**(b) YopO S585C/Q603C**	g = (2.0036, 2.0058, 2.0005)<br>A = 31.1 MHz<br>LWPP = (0, 0.031) mT<br>$\tau \approx$ 11 ns
**(c) free TSL 9**	g = 2.0034<br>$A_{1Phenyl}$ = 31.3 MHz<br>$A_{2,6Phenyl}$ = 25.3 MHz<br>$A_{3,5Phenyl}$ = 6.8 MHz<br>$A_{4Phenyl}$ = 9.4 MHz<br>LWPP = (0.017, 0.018) mT

8.3 Relaxation Time Measurements

Relaxation times T_1 and T_m were measured with the Inversion Recovery (IR) pulse sequence (Figure S19a) and via a two-pulse Electron Spin Echo Envelope Modulation (2pESEEM) experiment (Figure S19b). The pulse sequences were applied at the maximum of the field sweep spectrum and the temperature was set to 50 K, 60 K, 70 K and 80 K. Both IR and 2pESEEM experiments included phase cycling, two steps for 2pESEEM and four steps for IR.[4] All parameters of the IR and 2PESEEM pulse sequences are given in Table S4.

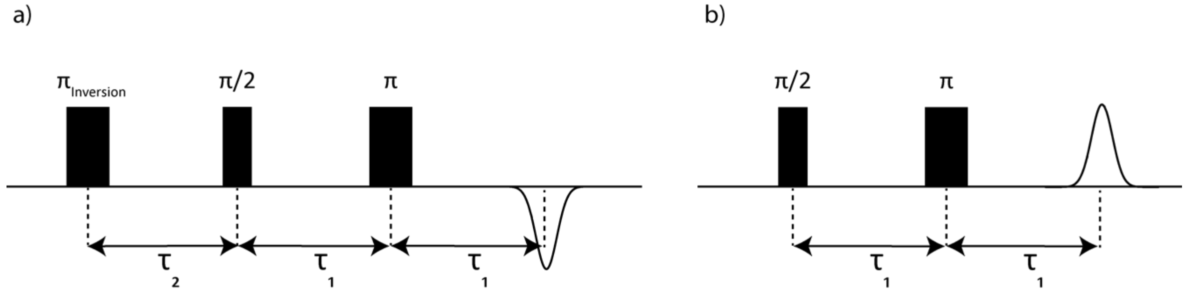

Figure S19. a) Inversion Recovery (IR) for T_1 measurements and b) two-Pulse ESEEM (2pESEEM) for T_m measurements.

Table S4. Pulse sequence parameters for the relaxation experiments.

**Inversion Recovery**		**Two-Pulse ESEEM**	
**Variable**	**Value**	**Variable**	**Value**
$\pi/2$	12 ns	$\pi/2$	12 ns
π	12 ns	π	24 ns
$\pi_{Inversion}$	22–24 ns	–	–
τ_1	300 ns	τ_1	200 ns
τ_2	400 ns	–	–
τ_2 increment	100 µs	τ_1 increment	8 ns
Shots per Point	10	Shots per Point	10
Shot Repetition Time	50 ms	Shot Repetition Time	40 ms

The T_1 relaxation times were extracted by multiplying the recorded IR curves by –1 and fitting a single exponential decay ($y = a \cdot \exp(-x/T_1) + c$).[12] The T_m relaxation times were obtained by fitting a stretched exponential decay ($y = a \cdot \exp((-x/T_m)^c) + d$) to the echo decay curves acquired by the 2pESEEM experiment.[4] The traces recorded from double mutant YopO V599**T9**/N624**T9** are displayed in Figure S20 and the fit values for T_1 and T_m are summarized in Table S5.

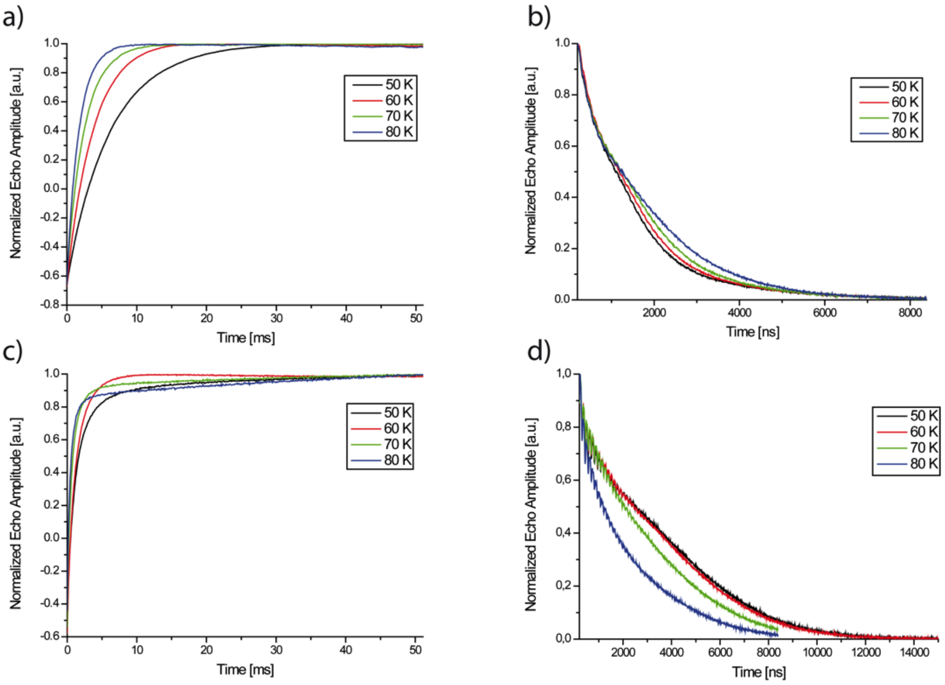

Figure S20. Inversion Recovery traces (a,c) and Two-Pulse ESEEM traces (b,d) recorded on V599**T9**/N624**T9** (a,b) and V599**R1**/N624**R1** (c,d) at different temperatures.

Table S5. Relaxation times of V599**T9**/N624**T9** and V599**R1**/N624**R1** at different temperatures.

	*V599**T9**/N624**T9***		*V599**R1**/N624**R1***	
**T [K]**	**T_1 [ms]**	**T_m [µs]**	**T_1 [ms]**	**T_m [µs]**
50	6.3	1.3	1.9	4.6
60	3.6	1.4	1.4	4.4
70	2.5	1.6	0.9	4.6
80	1.7	1.6	0.7	1.5

9. PDS Measurements

9.1 DQC

The six-pulse DQC sequence (Figure S21) was applied at the magnetic field position which yielded the maximal intensity in the field-swept EPR spectrum. The phase of the microwave radiation was adjusted such that the intensity of the DQC echo was maximal in the real channel. Pulse lengths and interpulse delays are given in Table S6. The shot repetition time (SRT) was set to 15.3 ms.[5] A 64–step phase cycle was applied to remove undesired echoes and thus extract the pure double quantum coherence pathway contributions.[6,7] In order to remove deuterium ESEEM from the dipolar traces, a modulation averaging procedure was applied (τ_1 and τ_2 in 8 steps of 16 ns).[2]

Table S6. Pulse sequence settings for DQC.

Variable	Value
$(\pi/2)_x$	12 ns
$(\pi/2)_y$	12 ns
π	24 ns
τ_1	250 ns
τ_2	4500 ns
T	50 ns
Shots per Point	3
Shot Repetition Time	15.3 ms

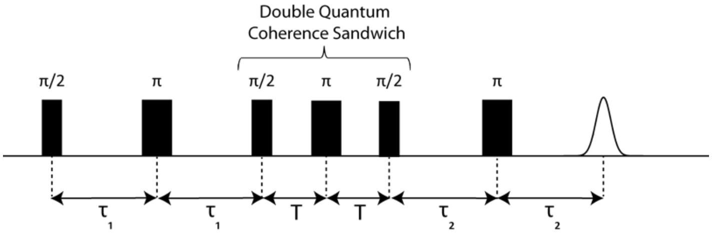

Figure S21. Schematic representation of the DQC pulse sequence. The employed sequence was adapted from literature procedures.[6]

9.2 SIFTER

The SIFTER sequence (Figure S22) in conjunction with a 16-step phase cycle[8] was applied at the magnetic field position yielding the highest signal amplitude in the field sweep spectra. Modulation averaging (τ_1 and τ_2 in 8 steps of 16 ns) was applied to remove deuterium ESEEM from the time traces. All pulse lengths, interpulse delay times and further parameters are given in Table S7.

Table S7. Pulse sequence settings for SIFTER.

Variable	Value
$(\pi/2)_x$	12 ns
$(\pi/2)_y$	12 ns
π	24 ns
τ_1	300 ns
τ_2	4500 ns
Shots per Point	3
Shot Repetition Time	15.3 ms

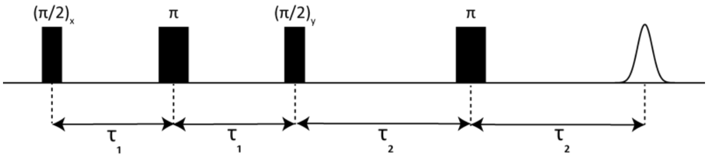

Figure S22. Schematic representation of the SIFTER pulse sequence. The employed sequence was adapted from literature procedures.[9]

9.3 PELDOR

For the PELDOR experiment (Figure S23)[9] on **9** the settings in Table S8 were used. The length of the pump pulse $(\pi)_B$ was determined by a transient nutation experiment. The pump pulse $(\pi)_B$ was set to the maximum of the field sweep spectrum and the observer pulses were applied at a frequency offset of –15 MHz relative to the pump frequency. Regarding the suppression of deuterium ESEEM, an 8-step modulation averaging procedure was applied with a time increment of 16 ns. Additionally, a two-step phase cycle was used in order to remove undesired echoes and to correct for receiver baseline offsets.

Table S8. Pulse sequence settings for PELDOR.

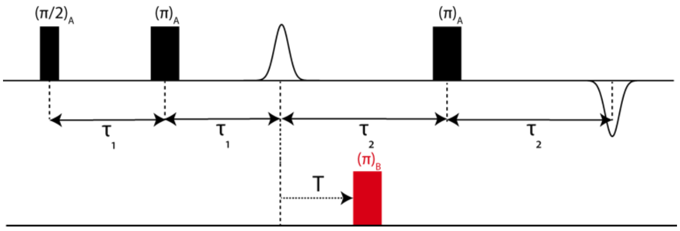

Figure S23. PELDOR pulse sequence.

Variable	Value
$(\pi/2)_A$	32 ns
$(\pi)_A$	64 ns
$(\pi)_B$	64 ns
τ_1	260 ns
τ_2	4500 ns
Shots per Point	3
Shot repetition time	15.3 ms

For the PELDOR experiment on **R1**-labeled YopO, the pump pulse was applied at the magnetic field position, which yields the maximal signal amplitude. The detection sequence was applied at a frequency offset of –100 MHz with respect to the pump frequency. The other parameters were set as given in Table S9. The optimal length of the $(\pi)_B$ pump pulse was determined by a transient nutation experiment. As mentioned above, a modulation averaging procedure and a two-step phase cycle was used to average out deuterium ESEEM and to remove unwanted echoes as well as baseline offsets.

Table S9. Pulse sequence settings for PELDOR on **R1**.

Variable	Value
$(\pi/2)_A$	12 ns
$(\pi)_A$	24 ns
$(\pi)_B$	16 ns
τ_1	300 ns
τ_2	4500 ns
Shots per Point	3
Shot Repetition Time	3.06 ms

9.4 Original PDS Time Traces, Background Removal and Validation

All PDS data was analyzed using the DeerAnalysis 2018 package for MATLAB.[10] In PDS, the resulting time trace is a convolution of the wanted dipolar interaction between the pair of spin labels within one protein molecule (intramolecular) and a background contribution between spins located on different macromolecules (intermolecular). The intramolecular dipolar interaction can be extracted by different procedures depending on the respective experiment: for PELDOR spectroscopy, the background is usually fitted directly to the time trace assuming a three-dimensional distribution of background nano-objects.[11,12] For the single-frequency experiments DQC and SIFTER, however, such an analytical treatment of the background is not applicable.[12,13] In this case, experimental background data obtained by performing DQC/SIFTER measurements on labeled single cysteine mutants have been used.[14-16] Then, an 8th order polynomial was fit to the thus obtained time traces (Figure S24) quantifying the experimental background. The DQC and SIFTER time traces were then

divided by these fits.[14-16] Figure S25-S26 display the datasets for PELDOR, DQC and SIFTER with background correction and validation for V599**T9**/N524**T9** and S585**T9**/Q603**T9**.

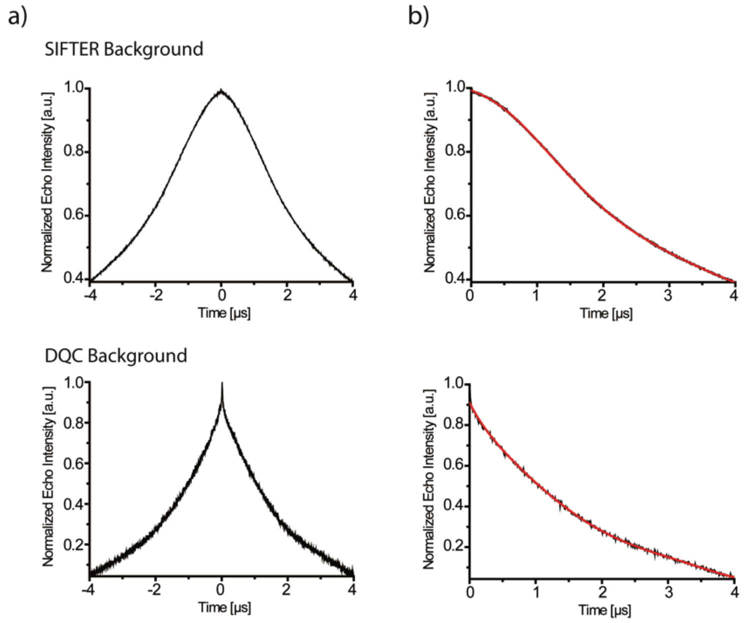

Figure S24. DQC and SIFTER time traces obtained from a YopO L113**T9** single mutant used for background correction. Subfigures (a) show the background traces prior to mirroring. In subfigure (b), the traces are mirrored at the zero-time origin and the red line indicates a polynomial fit of 8th order.

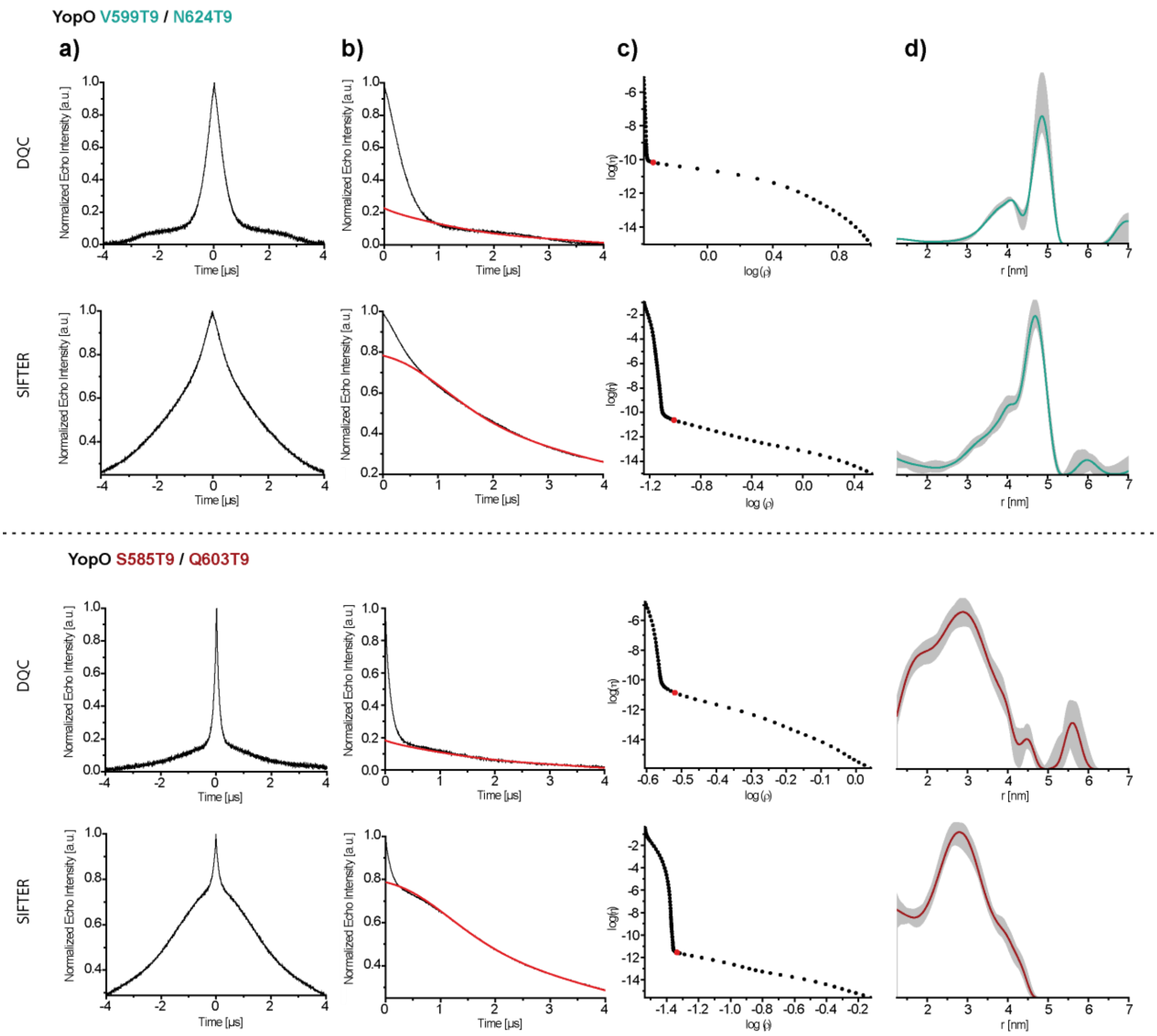

Figure S25. DQC and SIFTER time traces obtained from the YopO double mutants V599**T9**/N624**T9** and S585**T9**/Q603**T9**. Raw dipolar traces are shown prior to (a) and after (b) mirroring at the zero-time origin. The red line in panels (b) represents the experimental background fit obtained from the single cysteine mutant. Panels (c) show the L-curves generated by DeerAnalysis where the red dot marks the regularization parameter automatically chosen by DeerAnalysis for computing the distance distributions shown in panels (d).

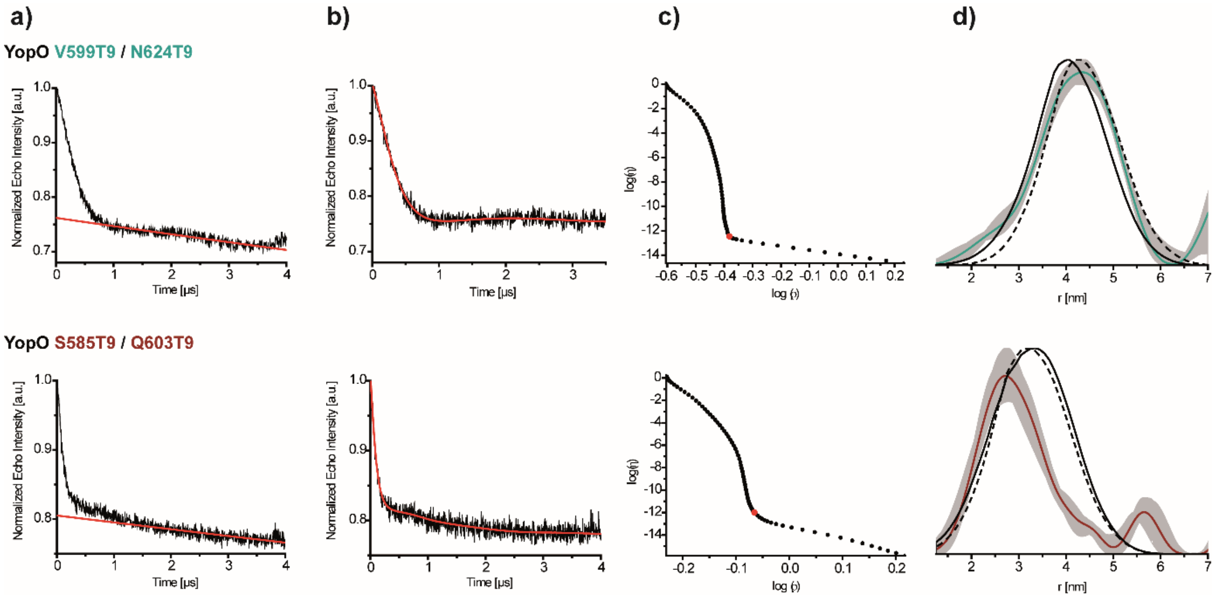

Figure S26. PELDOR time traces obtained from YopO double mutants V599**T9**/N624**T9** and S585**T9**/Q603**T9**. Panels (a) show the experimental time traces with a red line indicating the background fit assuming a three-dimensional homogeneous distribution of background objects. Column (b) displays the background-corrected dipolar traces with red lines indicating a fit used to compute distance distributions. Panel (c) shows the L-curves and the optimal regularization parameters determined by the L-curve criterion (c) which are used to compute the distance distributions shown in (d). The grey shaded areas in subfigures (d) indicate the uncertainty of the distance distributions as obtained by the Validation tool of DeerAnalysis and the black dashed and solid lines show the mtsslWizard predictions obtained from the PDB structures 4ci6 (dashed) and 2h7o (solid).

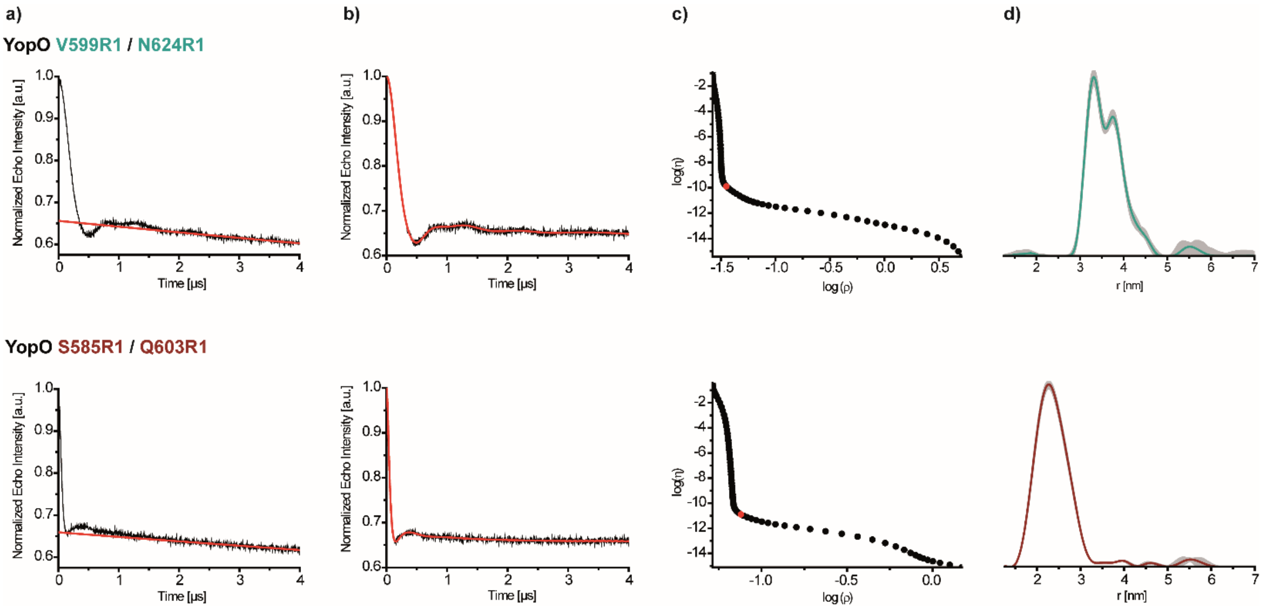

Figure S27. PELDOR data of YopO V599**R1**/N624**R1** (top) and S585**R1**/Q603**R1** (bottom). (a) Raw dipolar traces (black) and background fits (red). (b) Background-corrected time traces. (c) L-curves and the regularization parameter chosen for computing the distance distributions marked in red. (d) Distance distributions obtained by Tikhonov regularization.

9.5 Signal to Noise Ratio (SNR) determination

The quality of the recorded dipolar traces can be estimated by the signal-to-noise ratio (SNR) defined as

$$SNR = \frac{\lambda}{\sigma_N} \cdot \frac{1}{\sqrt{t}}$$

where λ is the modulation depth of the dipolar trace, t is the acquisition time of the respective experiment, and σ_N is the standard deviation of the noise of the trace. In order to deconvolve the noise from the wanted signal, the signal has been approximated by a polynomial fit (polynomial of second to 5th order). Subtracting this fit from the measured traces yields the pure noise contributions. The SNR has been calculated from the raw data prior to background-correction using the software SnrCalculator.[17] The thus obtained SNR values of all dipolar traces shown either in the main text or the supporting information are compiled in Table S10.

Table S10. Signal-to-Noise ratios obtained via different PDS measurements on trityl and nitroxide-labeled YopO double mutants.

	V599**Label**/N624**Label**	S585**Label**/Q603**Label**
DQC (**T9**)	8.9 min$^{-1/2}$	7.0 min$^{-1/2}$
SIFTER (**T9**)	5.8 min$^{-1/2}$	5.9 min$^{-1/2}$
PELDOR (**T9**)	1.4 min$^{-1/2}$	1.1 min$^{-1/2}$
PELDOR (**R1**)	9.9 min$^{-1/2}$	7.3 min$^{-1/2}$

10. Protein Work

10.1 Mutagenesis

Truncated YopO$_{89-729}$ C219A (YopO-WT) from *Yersinia enterocolitica* was cloned in frame into a pGex6p1 vector (GE) and amplified in *E. coli* DH5α cells. YopO V599C/N624C and YopO S585C/Q603C were constructed starting from YopO-WT by *QuickChange* mutagenesis[18] and subsequently transformed in *E. coli* DH5α cells. The employed primer pairs are declared in Table S11.

Table S11. Quick change mutagenesis primer pairs.

	Sequence
C219A *fwd*	5‘-GTGCTTCTGACACACTAAGAAGCCTCGCCGATAG-3‘
C219A *rev*	5‘-AGTGTGTCAGAAGCACGCCAACCATCCACCTC-3‘
V599C *fwd*	5‘-GCTTCCTGAATCGATTAGCTGAGGCTAAGTGCACCTTG-3‘
V599C *rev*	5‘-GGAGAGTATTCAATTGCTGCGACAAGGTGCACTTAGCC-3‘
N624C *fwd*	5‘-GAGAGTGCGAAAGCGCAACTATCTATTCTGATTTGTCGTTCAG-3‘
N624C *rev*	5‘-GAGCAACATCAGCCCAAGAACCTGAACGACAAATCAGAATAG-3‘
S585C *fwd*	5‘-CACAGCAAGGGCAGCCCGTGTCCTGTGAAACCT-3‘
S585C *rev*	5‘-CTAATCGATTCAGGAAGCTGTAGGTTTCACAGGACACG-3‘
Q603C *fwd*	5‘-CGATTAGCTGAGGCTAAGGTCACCTTGTCGTGTCAATTG-3‘
Q603C *rev*	5‘-CTGCTGCTGCTGGAGAGTATTCAATTGACACGACAAGG-3‘

After plasmid amplification, the mutagenesis was confirmed via Sanger sequencing (Figure S28).

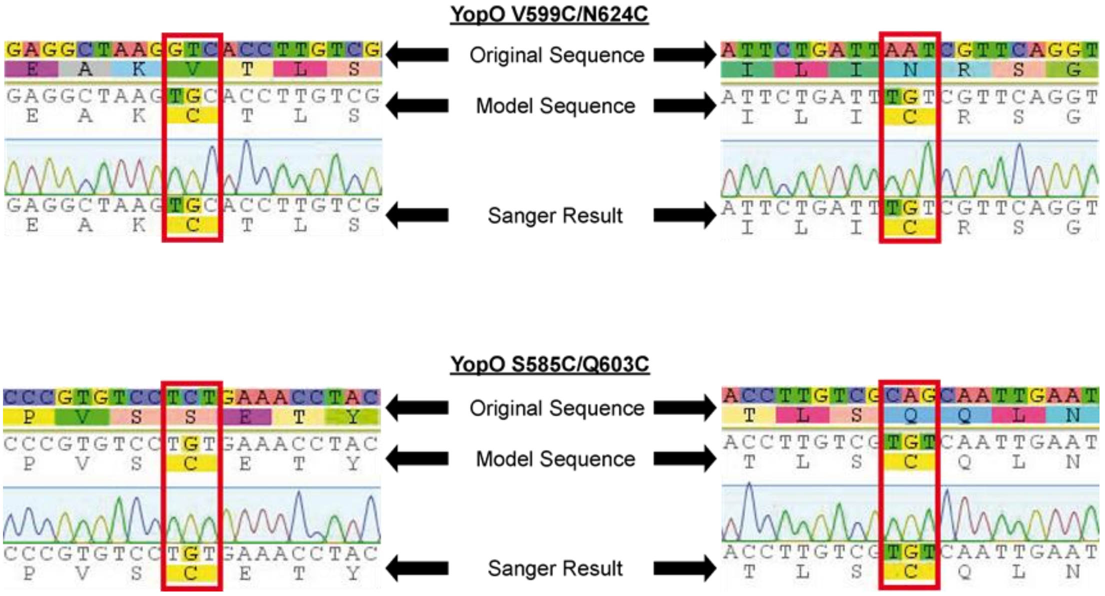

Figure S28. Sequencing results of YopO V509C/N624C and YopO S585C/Q603C.

10.2 Expression and Purification

All YopO constructs were expressed in *E. coli* Rosetta (DE3) cells. The cells were cultured in LB medium containing 0.3 mM ampicillin and 0.1 mM chloramphenicol for selection purposes. Cell cultures were incubated at 37 °C until an OD_{600} of ~0.8-1.0 was reached. Protein expression was induced with 0.1 mM IPTG (isopropyl β-D-1-thiogalactopyranoside), the culture was further incubated for ~16 h at 16 °C and then, the pellet was harvested after separation by centrifugation (4000 rcf, 20 min, 4 °C).

The cell pellet was re-suspended in five-times w/v lysis buffer (50 mM Tris•HCl pH 8.0, 150 mM NaCl and 3 mM DTT) and lysed twice at 30 kpsi in a cell disruptor (Constant Systems Limited) The lysate was spun down to remove insoluble cell debris (48,500 rcf, 20 min, 4 °C) and the supernatant was incubated with GST sepharose (GE) beads which were then equilibrated with lysis buffer for 1 h at room temperature under slight agitation. The GST-suspension was filled into a gravity column and the flowthrough was run over the column an additional time to increase the total protein yield. The beads were washed with 50 mL lysis buffer and the protein elution from the GST beads took place overnight in 20 mL elution buffer (50 mM Tris•HCl pH 8.0, 150 mM NaCl, 1 mM DTT, 1mM EDTA, 100 U PreScission protease) at 4 °C stimulated by gentle shaking. The flowthrough was diluted with 150 mL no-salt buffer (50 mM Tris•HCl pH 8.0) and 220 µL of a 2 M DTT stock were added. An ion-exchange chromatography against a linear gradient of high-salt buffer (50 mM Tris•HCl pH 8.0, 1 M NaCl) was performed using an EnrichQ 10/100 column (Bio-Rad) and the fractions containing pure YopO (based on SDS-PAGE and subsequent Coomassie stain) were pooled and concentrated below 3 mL using a centrifugal concentrator. The sample was further purified via size-exclusion chromatography in gel filtration buffer (50 mM Tris•HCl pH 8.0, 50 mM NaCl) on a HiLoad Superdex 200 16/600 (GE Healthcare), the fractions containing the target protein (Figure S29) were pooled and concentrated to ~100 µM YopO. The samples were flash frozen and stored in 50 µL aliquots at -80 °C. From 1 L cell culture, ~ 1.4 mg protein were obtained.

YopO-WT

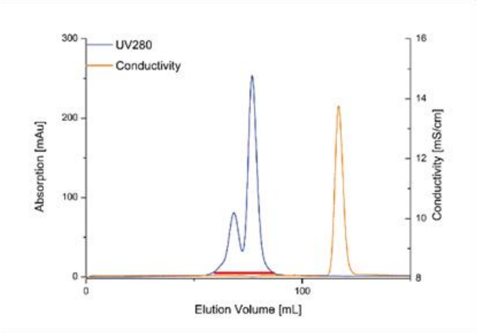

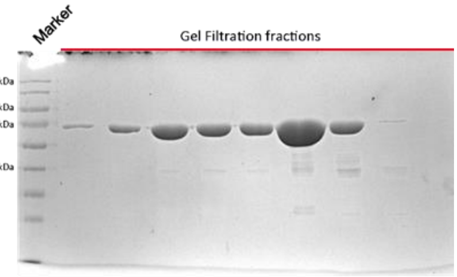

YopO-V599C/N624C

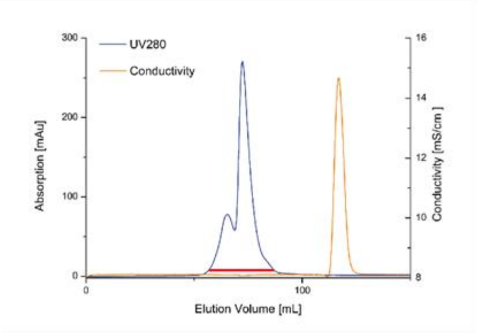

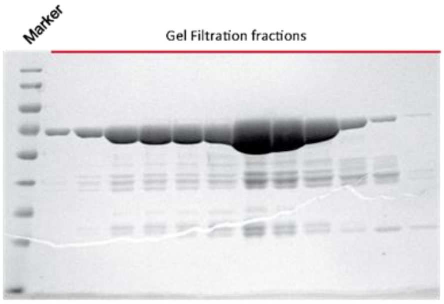

YopO-S585C/Q603C

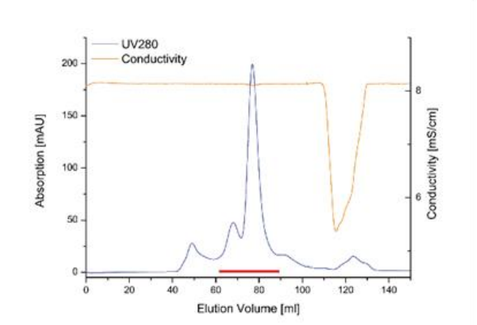

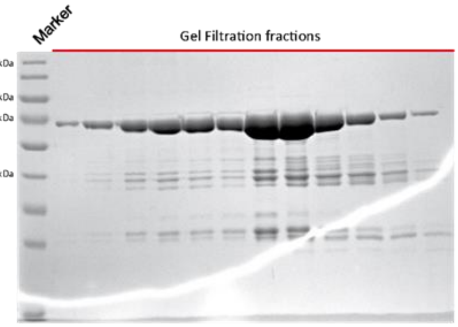

Figure S29. Chromatograms of the final HiLoad Superdex 16/600 200pg runs for all YopO constructs used (left). SDS-PAGE gels of the marked (red) elution fractions (right).

11. References

[1] Stoll, S., Schweiger, A. (2006), J. Magn. Reson. 178, 42–55.

[2] Akhmetzyanov, D., Schöps, P., Marko, A., Kunjir, N. C., Sigurdsson, S. Th., Prisner, T. F. (2015), Phys. Chem. Chem. Phys. 17, 24446–24451.

[3] Bowman, M. K. Mailer, C., Halpern, H. J. (2005), J. Magn. Reson. 172, 254–267.

[4] Weber, R. T. (2005) ELEXSYS E580 Pulse EPR Spectrometer User's Manual, Chapter 6, Bruker BioSpin Corporation, USA.

[5] Stoll, S. (2018) EPR Spectroscopy: Fundamentals and Methods (Goldfarb, D., Stoll, S., Eds.), Chapter 11, Wiley, UK.

[6] Borbat, P. P., Freed, J. H. (1999) Chem. Phys. Lett. 313, 145–154.

[7] Saxena, S., Freed, J. H. (1997) J. Chem. Phys. 107, 1317–1340.

[8] Jeschke, G., Pannier, M., Godt, A., Spiess H. W. (2000) Chem. Phys. Lett. 331, 243–252.

[9] Meyer, A., Jassoy, J. J., Spicher, S., Berndhäuser, A., Schiemann, O. (2018) Phys. Chem. Chem. Phys. 20, 13858-13869.

[10] Jeschke, G., Chechik, V., Ionita, P., Godt, A., Zimmermann, H., Banham, J., Timmel, C. R., Hilger, D., Jung, H. (2006) Appl. Magn. Reson. 30, 473–498.

[11] Milov, A. D., Maryasov, A. G., Tevetkov, Y. D. (1998) Appl. Magn. Reson. 15, 107–143.

[12] Jeschke, G. (2014) Struct. Bond. 152, 83–120.

[13] Borbat, P. P., Mchaourab, H. S., Freed, J. H. (2002) J. Am. Chem. Soc. 124, 5304–5314.

[14] Akhmetzyanov, D., Schöps, P., Marko, A., Kunjir, N., Sigurdsson, S. Th., Prisner, T. F. (2015) Phys. Chem. Chem. Phys. 17, 24446–24451.

[15] Yang, Z., Liu, Y., Borbat, P. P., Zweier, J. L., Freed, J. H., Hubbell, W. L. (2012) J. Am. Chem. Soc. 134, 9950–9952.

[16] Shevelev, G. Y., Krumkacheva, O. A., Lomzov, A. A., Kuzhelev, A. A., Rogozhnikova, O. Y., Trukhin, D. V., Troitskaya, T. I., Tormyshev, V. M., Fedin, M. V., Pyshnyi, D. V., Bagryanskaya, E. G. (2014) J. Am. Chem. Soc. 136, 9874–9877.

[17] https://github.com/dinarabdullin/SnrCalculator

[18] Liu, H., Naismith, J. H. (2008) BMC Biotechnol. 8, 91–101.
